# A versatile fluorinated azamacrocyclic chelator enabling ^18^F PET or ^19^F MRI: a first step towards new multimodal and smart contrast agents†

**DOI:** 10.1039/d4sc02871f

**Published:** 2024-07-25

**Authors:** Charline Sire, Vincent Meneyrol, Nathalie Saffon-Merceron, Enzo Terreno, Francesca Garello, Lorenzo Tei, Emmanuelle Jestin, Raphaël Tripier, Thibault Troadec

**Affiliations:** a Univ. Brest, UMR CNRS 6521 CEMCA 6 Avenue Victor Le Gorgeu 29200 Brest France Thibault.troadec@univ-brest.fr; b Cyclotron Réunion Océan Indien CYROI 2 rue Maxime Rivière 97490 Sainte-Clotilde France; c Institut de Chimie de Toulouse (UAR 2599), 118 route de Narbonne 31062 Toulouse Cedex 9 France; d Department of Molecular Biotechnology and Health Sciences, University of Turin Piazza Nizza 44/bis 10126 Turin Italy; e Dipartimento di Scienze ed Innovazione Tecnologica, Università del Piemonte Orientale “Amedeo Avogadro” Viale T. Michel 11 15121 Alessandria Italy

## Abstract

Macrocyclic chelators play a central role in medical imaging and nuclear medicine owing to their unparalleled metal cation coordination abilities. Their functionalization by fluorinated groups is an attractive design, to combine their properties with those of ^18^F for Positron Emission Tomography (PET) or natural ^19^F for Magnetic Resonance Imaging (MRI), and access potential theranostic or smart medical imaging probes. For the first time, a compact fluorinated macrocyclic architecture has been synthesized, based on a cyclen chelator bearing additional pyridine coordinating units and simple methyltrifluoroborate prosthetic groups. This ligand and its corresponding model Zn(ii) complex were investigated to evaluate the ^18^F-PET or ^19^F MRI abilities provided by this novel molecular structure. The chelator and the complex were obtained *via* a simple and high-yielding synthetic route, present excellent solvolytic stability of the trifluoroborate groups at various pH, and provide facile late-stage ^18^F-radiolabeling (up to 68% radiochemical yield with high activity) as well as a satisfying detection limit for ^19^F MRI imaging (low mM range).

## Abstract 2

A new trifluoroborate-appended macrocyclic chelator presents suitable ^19^F MRI signal and late-stage ^18^F-radiolabeling, paving the way for the design of novel theranostic and smart imaging agents.

## Graphical abstract

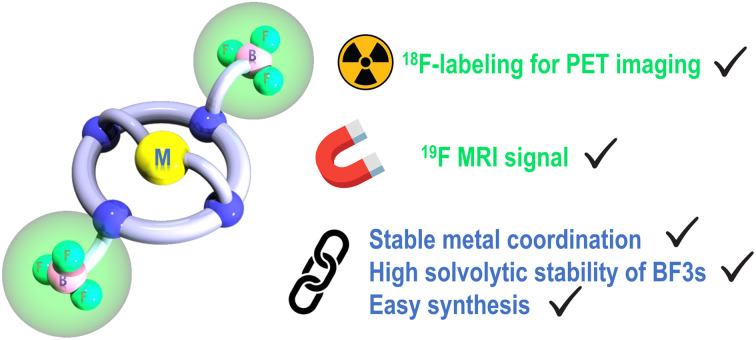

## Introduction

Fluorine is a ubiquitous element in medical imaging with its two main isotopes, natural ^19^F and radioactive ^18^F, that allow Magnetic Resonance Imaging (MRI) and Positron Emission Tomography (PET), respectively. On the one hand, ^18^F is the most used and readily available radionuclide for PET, with a wide range of radiolabeling strategies currently available,^1,2^ as it has the advantages of a biologically relevant radioactive half-life (*t*_1/2_ = 110 min), low radiotoxicity and low positron (β^+^) emission energy resulting in high resolution images. On the other hand, ^19^F-MRI presents the intrinsic advantages of the natural abundance of ^19^F (100%) and its good sensitivity compared to other nuclei (second best behind ^1^H used in MRI). Moreover, the lack of *in vivo* background owing to the absence of fluorine in the body soft tissues allows direct quantitative detection of the tracer, in opposition to conventional ^1^H MRI that relies on the water content of the body.^3,4^ However this also leads to a low overall sensitivity of the ^19^F MRI technique that is currently tackled by two distinct strategies when it comes to molecular probes: high fluorine content of the tracer (ex: perfluorinated alcanes)^4^ or combination with paramagnetic metals or lanthanides that lower the relaxation time of the sensors.^5^

Concurrently, in the field of molecular probes for medical imaging and therapy, saturated polyazamacrocycles, such as cyclen (1,4,7,10-tetraazacyclododecane), cyclam (1,4,8,11-tetraazacyclotetradecane) and tacn (1,4,7-triazacyclononane) are cornerstone for the complexation of metallic and lanthanide cations for MRI,^6^ PET,^7,8^ Single-Photon Emission Computed Tomography (SPECT)^9^ or internal Radiotherapy,^7,8^ owing to their exceptional coordination properties and *in vivo* inertness of their complexes. In that context, fluorinated azamacrocycles are highly attractive as they can combine, within a single molecular architecture, the properties of metals or radiometals coordinated within their cavity with the ones of ^19^F or ^18^F. In particular, two major applications have been targeted recently: (i) complexes combining radiometals (α or β^−^ emitters) for radiotherapy and prosthetic ^18^F-radiolabeled units for diagnosis, leading to so-called theranostic tracers that merge therapeutic and diagnostic modalities in a single molecule and provide identical biodistribution *in vivo*; and (ii) complexes of paramagnetic cations with natural ^19^F pendants as sensitive or responsive ^19^F MRI probes.

**Scheme 1:**
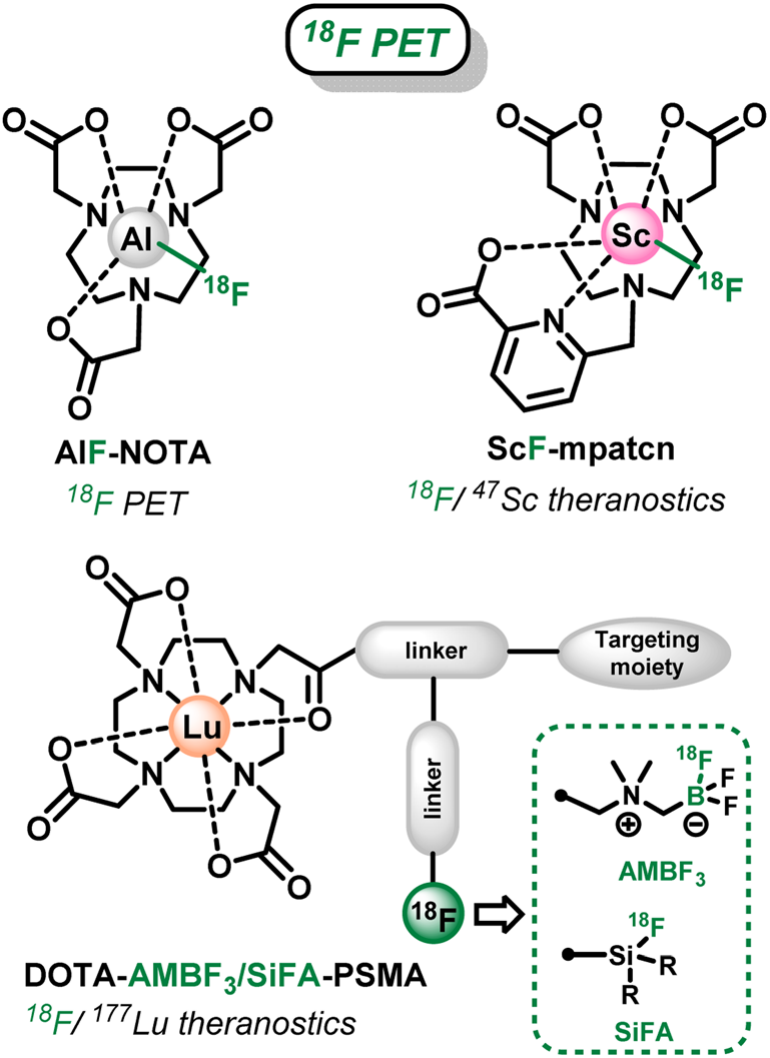
Fluorinated azamacrocyclic complexes for ^18^F-PET imaging and theranostics.

Several fluorinated polyazacycloalcane scaffolds have thus been described for these two distinct objectives in recent years. First, azamacrocycles were used in ^18^F-PET as an alternative to conventional ^18^F-labeling (*via* C–F bonds formation)^10,11^ for the tagging of biomolecules. In this case, the macrocyclic cavity of tacn-based chelators was used to accommodate metal-^18^F synthons: with Al(iii) over a decade ago (AlF-NOTA, Scheme 1),^12–14^ and lately with Ga(iii) and Fe(iii).^15–18^ Linear ligands, such as ResCa and analogues, have also been reported since to allow such radiolabeling at low temperature (<37 °C).^19–23^ However in those cases, the chelating cavity is occupied by innocent metal cations bearing no additional property. Only very recently Boros *et al.* went a step further and developed the corresponding theranostic version, using the Sc(iii)–F synthon that can allow the ^47^Sc (β^−^ radiotherapy)/^18^F (PET imaging) theranostic couple.^24^ Nevertheless, the scope of this strategy is limited in terms of possible metal cations and corresponding radiometals as the strength of the M–F interaction is not sufficient with most relevant metallic cations, as already observed for instance with lanthanides.^25,26^

In a second strategy, the well-known DOTA chelator ((1,4,7,10-tetraazacyclododecane-1,4,7,10-tetrayl)tetraacetic acid), that can coordinate a wide range of metal cations and ^177^Lu (β^−^ emitter) in particular, has been functionalized by branched side chains allowing grafting on a targeting moiety and installation of fluoroborate or fluorosilane prosthetic groups.^27,28^ These are particularly attractive in this context, as they allow easy late-stage ^18^F-labeling *via*^19^F/^18^F isotopic exchange processes (Scheme 1),^29–31^ as an alternative to sometimes tedious C–^18^F bond forming reactions.^10,11^ However, the organic frameworks attached to the chelator in this case require challenging multistep syntheses, and result in large branched structures that can be detrimental to the recognition properties of the targeting unit and biodistribution of the radiopharmaceuticals. Therefore, more compact architecture are highly desirable.

**Scheme 2:**
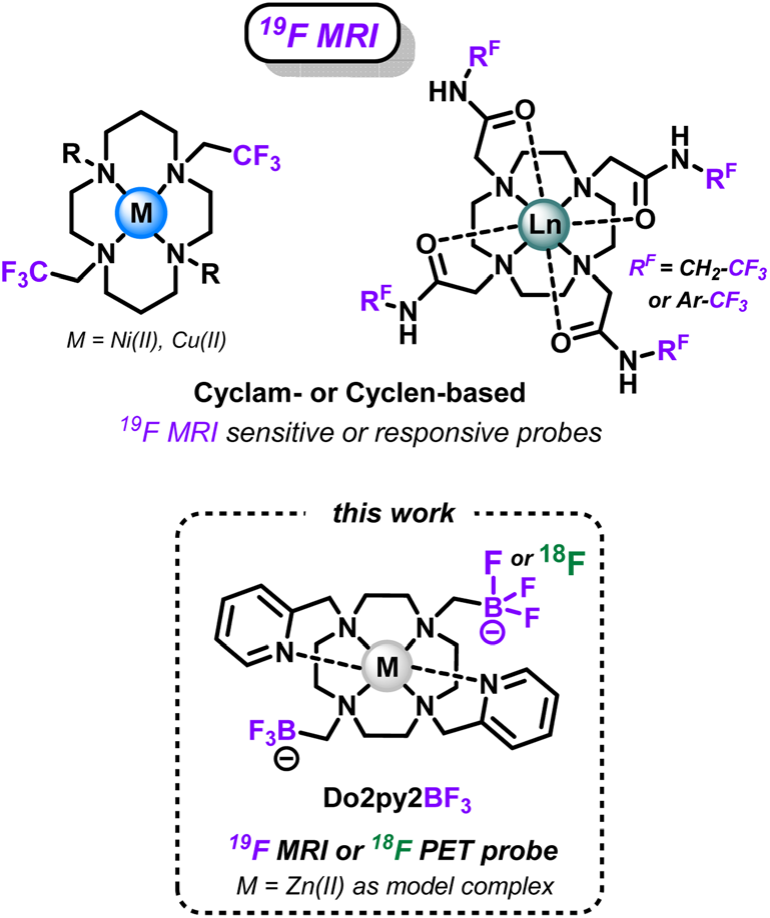
Fluorinated azamacrocyclic complexes as sensitive or responsive probes for ^19^F-MRI imaging, and versatile Do2py2BF_3_ ligand presented in this work.

Concerning the ^19^F MRI modality, small trifluoromethyl units have been efficiently used in sensitive or responsive probes based on azamacrocyclic complexes. First, trifluoromethylated cyclen-based chelators (Scheme 2) have proven excellent to coordinate paramagnetic lanthanide cations (Tb^3+^, Dy^3+^, Ho^3+^, Er^3+^ in particular) that decrease ^19^F relaxation times and drastically enhance the probes sensitivity.^5,32–34^ Similarly, CF_3_-appended cyclam chelators (Scheme 2) have been used with nickel(ii) cations as sensitive probes,^35,36^ or as on–off responsive sensors with redox-active cations (Co^2+^, Cu^2+^) that can modulate the ^19^F MRI signal upon modification of their oxidation state by external stimuli.^37,38^ However, the lipophilic nature of such trifluoromethylated groups can be detrimental to applications in aqueous media and *in vivo* in particular.^32^

Therefore, we report in this study a novel compact cyclen-based architecture, bearing methyltrifluoroborate units directly supported by the macrocyclic amines (Do2py2BF_3_, Scheme 2), to target two different applications with the same molecular topology: (i) allow easy ^18^F-radiolabeling *via*^19^F/^18^F isotopic exchange to allow future theranostic combinations with relevant radiometals; and (ii) present suitable ^19^F MRI signal, which has never been explored in the case of trifluoroborates, that could be used in responsive or sensitive imaging agents upon coordination with relevant non-radioactive paramagnetic cations. Noteworthy in the field (see AlF-NOTA or ScF-mptacn, Scheme 1), the chemistry of such chelators is first developed without the introduction of grafting functions and targeting molecules that will be necessary for future *in vivo* targeted applications. Therefore, the bispyridyl-cyclen scaffold has been selected in this proof-of-concept study for its ease of preparation and ubiquitous coordination properties.^39–41^ Herein, the synthesis and characterization of this novel ligand architecture is presented, as well as the corresponding zinc(ii) complex, a convenient diamagnetic model complex that allowed easy NMR characterizations and simple stability studies. The ability of the scaffold to undergo ^18^F-labeling, as known for other types of trifluoroborates, was investigated on this new structure. Then the potential of BF_3_ units as ^19^F MRI reporters was also investigated for the first time on the model Zn(ii) complex. Finally, a particular focus was given to the study of the solvolytic stability of the new trifluoroborated scaffolds in water at different pH, which is a crucial parameter to envisage future applications for this new type of chelators.

## Results & discussion

### Synthesis and characterization

**Scheme 3:**
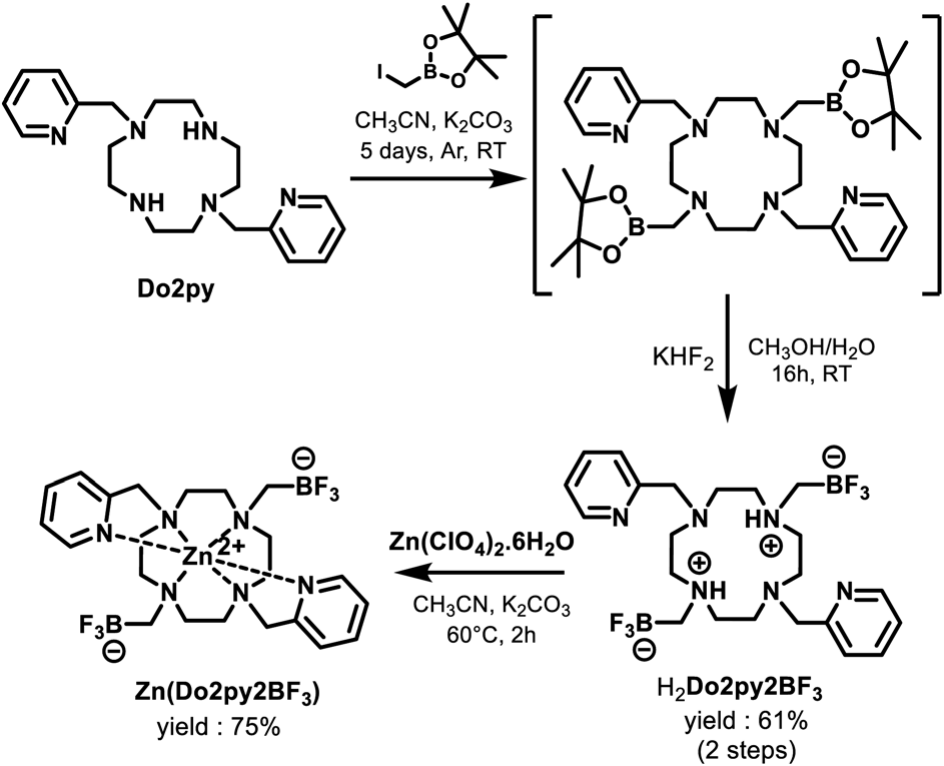
Synthesis of H_2_Do2py2BF_3_ ligand and Zn(Do2py2BF_3_) complex.

Ligand H_2_Do2py2BF_3_ was prepared from previously described Do2py,^40^ through a 2-step synthetic procedure adapted from Perrin *et al.* for the installation of methyltrifluoroborate functional groups *via* fluorination of a pinacolborane intermediate with potassium bifluoride (Scheme 3).^42^

**Fig. 1:**
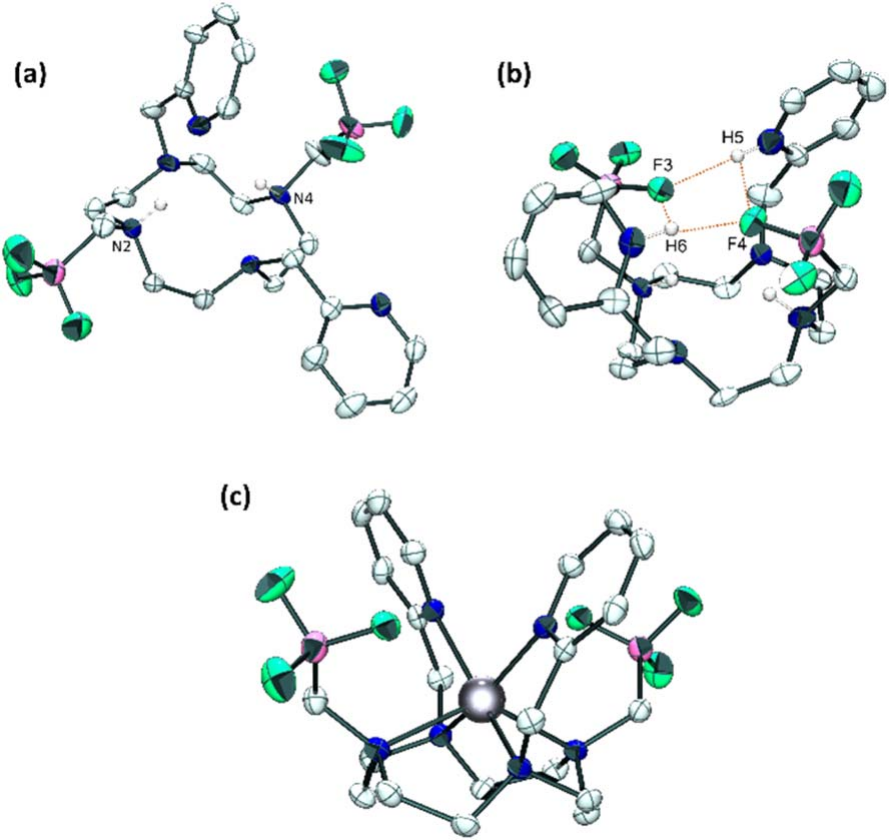
X-Ray diffraction structures of (a) H_2_Do2py2BF_3_ ligand at pH 4, (b) H_4_Do2py2BF_3_ at pH 1 and (c) Zn(Do2py2BF_3_) complex (c). Ellipsoids are drawn with 50% probability. H atoms omitted for clarity, except on ammonium groups of H_2_Do2py2BF_3_ and H_4_Do2py2BF_3_.

The new macrocycle was fully characterized by means of High-Resolution Mass Spectrometry (HRMS) and multinuclear NMR. In 1 : 1 D_2_O/CD_3_CN (pH 4), the ligand exhibits broad singlets in ^19^F (*δ* = −135.6 ppm) and ^11^B (*δ* = 2.44 ppm) NMR and a diagnostic quadruplet for the α-methylene group in ^1^H NMR (*δ* = 1.80 ppm, ^3^*J*_H–F_ = 5.2 Hz) (see ESI† for spectral data). In ^13^C NMR, this methylenic carbon could not be directly observed owing to multiplicity and broadness induced by neighboring boron and fluorine nuclei, but was assigned thanks to 2D ^1^H–^13^C HSQC NMR (47.8 ppm). Single crystals were grown from a water/acetonitrile mixture (1 : 1, pH 4) revealing the neutral H_2_Do2py2BF_3_ ligand with both nitrogen atoms holding the BF_3_ pendants (N2 and N4, Fig. 1a) that are protonated.^43^ This feature is classical for cyclen-based ligands at this pH, as two amines in *trans* N1–N3 positions generally have p*K*_a_s above 9.^44^

Single crystals were also grown from an acidic aqueous solution (pH 1), revealing a different structure with the ligand under the form H_4_Do2py2BF_3_^2+^ displaying additional protonation of the two pyridine units (H5 and H6, Fig. 1b) creating a H-bonding network with fluorine atoms (F3 and F4) from both trifluoroborate pendants (*d*_F4–H5_ and *d*_F3–H6_ of 1.89 and 1.91 Å respectively). This constrained geometry is also present in solution at acidic pH, as evidenced by a set of 3 broad signals on the ^19^F spectrum from −136 to −140 ppm (D_2_O, pH 1), close to the sole chemical shift observed at pH 4 (−135.6 ppm). Similarly, ^1^H NMR spectrum reveals the different symmetry of the molecule with a complex multiplet centered at 4.16 ppm accounting for the four methylenic pyridyl protons (*vs.* sharp singlet at 4.49 ppm at pH 4), different multiplicity of the aromatic protons and a set of three broad signals between 1.90 and 2.30 ppm for the methylenic protons linked to the trifluoroborate units.

The corresponding zinc(ii) complex was then prepared, from an equimolar mixture of H_2_Do2py2BF_3_ and zinc(ii) perchlorate in acetonitrile at 60 °C, with potassium carbonate as a base, and purified by crystallization in a water/acetonitrile mixture (3 : 7). Owing to its limited solubility in water at high concentrations, NMR analysis was carried out in a 1 : 1 D_2_O/CD_3_CN mixture (pH 6). At RT, the ^1^H spectrum presents very broad signals for the macrocyclic protons, that could however be resolved at 333 K (Fig. S11, ESI†), and ^19^F NMR reveals a broad singlet at −137.4 ppm, very close to that of free ligand, as a first indication of the innocent nature of the trifluoroborate pendants upon coordination of the metal. More insight was gained by the structure revealed by X-ray diffraction analysis on single crystals of the complex grown in CH_3_CN/H_2_O (7 : 3) solution (Fig. 1c). Zinc(ii) cation lies slightly above the cyclen plane (distance with N1–N2–N3–N4 centroid = 0.999 Å, Fig. S13, ESI†) and adopts a distorted trigonal prismatic geometry similar to the Zn(Do2py)^2+^ complex reported in the literature,^40^ highlighting again the innocent behaviour of the trifluoroborated groups in the coordination sphere of the metal. Only the Zn–N bonds with cyclen nitrogen atoms are somewhat longer in this complex (by up to 0.07 Å, Fig. S13, ESI†), probably owing to the different steric bulk on two nitrogen atoms from the ring (N–CH_2_–BF_3_*vs.* NH in Do2py).

### Solvolytic stability

The main limitation of trifluoroborate prosthetic groups for practical use in [^18^F]-PET radiotracers can be their lack of stability towards solvolysis, *via* the release of free fluoride and formation of boronic acid derivatives, which has been well-documented by Perrin *et al.*^45,46^

The solvolytic stability of the new azamacrocyclic motifs was thus investigated for the free ligand and the Zn complex by ^19^F NMR, at a concentration of 3 × 10^−2^ M in buffered aqueous solutions at pH 2.0, 7.3 and 9.7 (Fig. S15–S20, ESI†). In these conditions, Do2py2BF_3_ demonstrated exceptional robustness with no degradation observed in any of the three pH conditions, after up to 3 days at 25 °C (Table 1). In particular, this observation is in contrast with the closest reported ammonium-based structure (Et_2_NH^+^CH_2_BF_3_) that displays a half-life of 66 hours at physiological pH.^46^ This enhanced stability in Do2py2BF_3_ is probably due to the higher basicity of the cyclen tertiary amines providing a poorly labile ammonium proton stabilizing the zwitterionic structure with the trifluoroborate group. On the zinc(ii) complex, the stability is slightly different, owing to the loss of the ammonium protons to accommodate the metallic cation. First, at pH 2.0, a fast decoordination of the cation was evidenced by ^19^F NMR (*t*_1/2(*Z*n)_ < 5 min, Fig. S18, ESI†), which is common with such highly basic polyamines at acidic pH. However, no degradation of the BF_3_ units was observed as only the ^19^F signals of the free ligand were recovered. At neutral and basic pH, no release of the metal occurred, demonstrating the strong coordination ability of the ligand. However, very slow solvolysis of the BF_3_ groups appeared (Fig. S19–S20,† ESI). As previously discussed in the literature by Perrin and others,^45,47^ the kinetics of solvolysis follow a pseudo-first order kinetic rate, with a rate-determining step corresponding to the loss of the first fluoride ion, and the other intermediates towards fully hydrolyzed boronic acid being fast-lived. Very long half-lives (985 and 400 hours at pH 7.3 and 9.7 respectively) could be calculated here for the new BF_3_ pendants, that are perfectly suited to consider further use in the context of ^18^F PET imaging when compared to the radioactive half-life of ^18^F (110 min) or the biodistribution and clearance of radiotracers (from minutes to a few days). Indeed, the stability of these pendants even surpasses the one of the most stable similar trifluoroborate functions described so far in the literature in these pH ranges (366 h for AMBF_3_ groups at pH 7.5).

**Table 1:** Solvolysis data of Do2py2BF_3_ ligand and Zn(Do2py2BF_3_) complex

Compound	pH	*t*	% intact BF_3_	*t*_1/2_
Do2py2BF_3_	2.0	72 h	100%	—
	7.3	72 h	100%	—
	9.7	72 h	100%	—
Zn(Do2py2BF_3_)^2+^	2.0	8 days	100% (90% Zn release)	—
	7.3	22 h	89%	985 h
		13 days	77%	
	9.7	18 h	90%	400 h
		11 days	63%	

### ^18^F-radiolabeling

**Scheme 4:**
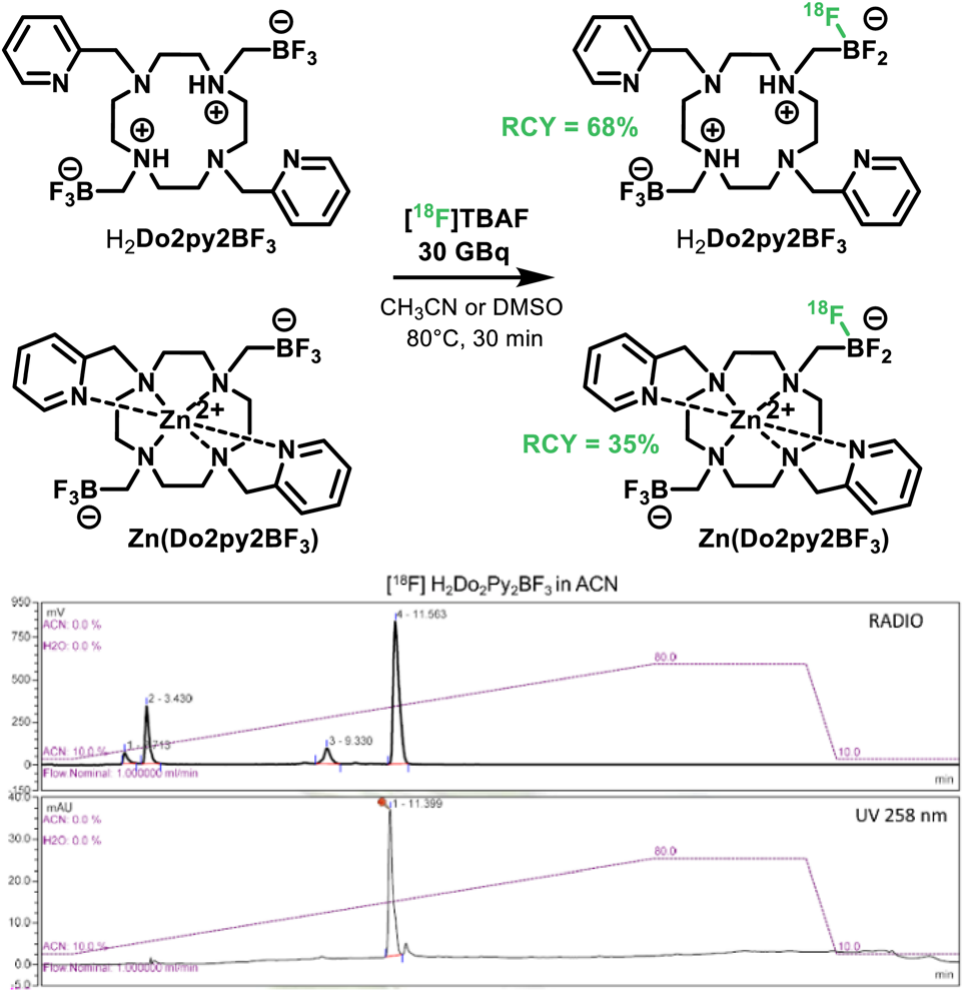
^18^F-radiolabeling of H_2_Do2py2BF_3_ and Zn(Do2py2BF_3_) in acetonitrile, and corresponding HPLC (UV) and radioHPLC traces for H_2_Do2py2BF_3_.

Furthermore the ability of the new molecule for easy ^18^F-radiolabeling was evaluated on both H_2_Do2py2BF_3_ ligand and Zn(Do2py2BF_3_) (Scheme 4). Radiosyntheses were carried out on a TRACERlab FXFN module (GE) (Fig. S21, ESI†). At the end of bombardement (EOB), the [^18^F] fluoride produced by the cyclotron was delivered to the automate and trapped on a pre-conditioned QMA Sep-Pak cartridge to remove [^18^O]–water. The elution of the activity (30 GBq at EOB) into the reactor was performed with a solution of TBABr (16 mg in 0.5 ml H_2_O) to form [^18^F]tetrabutylammonium fluoride ([^18^F]TBAF). Acetonitrile was added for the azeotropic evaporation under vacuum and helium flow. Solutions containing 1 mg of H_2_Do2py2BF_3_ in acetonitrile or Zn(Do2py2BF_3_) in DMSO were added into the reactor for a 30 min incubation at 80 °C. It is worth noting that these solvents are not desired for further application *in vivo*, but were used herein as the ligand and complex are not fully soluble in water. However, solubility in aqueous media should be achieved for future applications upon grafting on water soluble biomolecules, and aqueous ^18^F-labelling of trifluoroborates has been well documented.^48,49^

RadioChemical Yields (RCY) were subsequently measured *via* radioHPLC analyses, with UV traces of the “cold” ligand or zinc complex as references (Fig. S22–S23, ESI†). In these conditions, highly satisfactory RCY of up to 68% was obtained for the ligand H_2_Do2py2BF_3_. RCY for the Zn(Do2py2BF_3_) complex is somewhat lower, probably owing to its lower solubility in the reaction medium, but is acceptable to envisage future applications in the field. Indeed, these RCY are excellent when compared to analogous isotopic exchange with trifluoroborates in similar conditions (15–30%)^42,50^ or labeling *via* metal-fluoride synthons (30–45% with Al,^51^ 65% with Sc (ref. 24)). In addition to these conversions, labeled compounds could also be easily purified on semi-preparative HPLC with a Gemini C_18_ Column and formulated as injectable aqueous solutions after solubilization in NaCl solutions on C_18_ Sep-Pak cartridges within the automated system, which is one of the main advantages of such facile isotopic exchange on BF_3_ units. For the H_2_Do2py2BF_3_ radiolabeling, 30.6 GBq μmol^−1^ at the injection time were associated to the desired product whereas 7.8 GBq μmol^−1^ were obtained for the radiolabeled Zn(Do2py2BF_3_). These results on the molar activities are good enough for further *in vivo* studies and consistent with the reference publications (around 40–110 GBq μmol^−1^ for high specific activity).^48^

### ^19^F-magnetic resonance

**Fig. 2:**
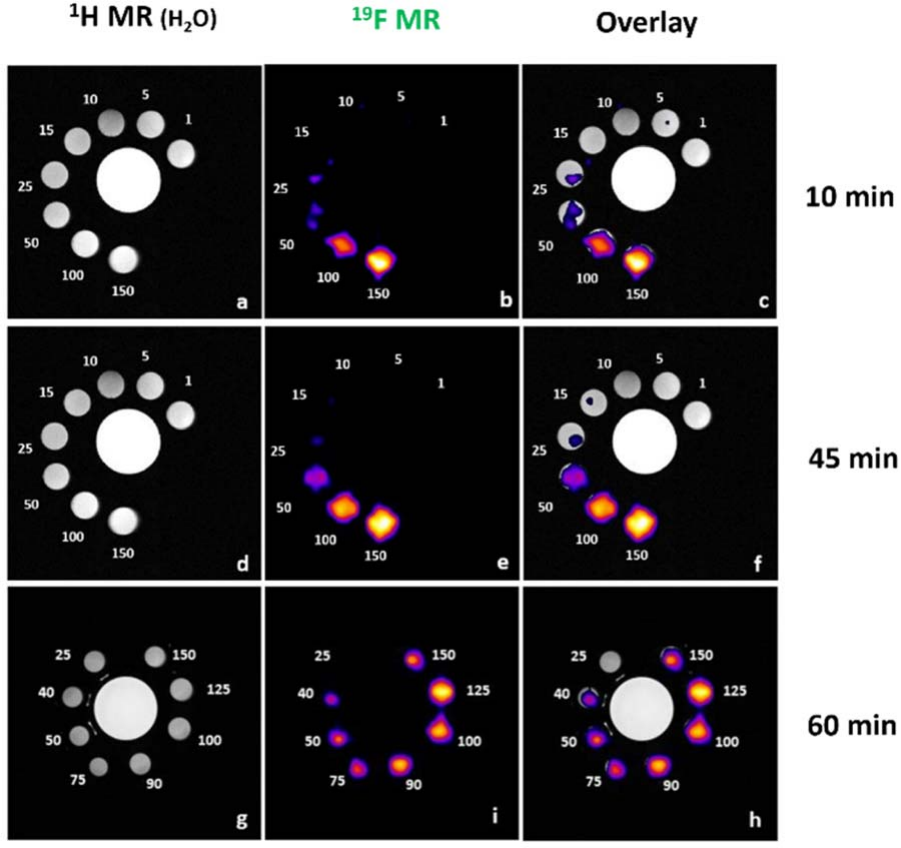
^19^F-Magnetic resonance of Zn(Do2py2BF_3_) in acetonitrile/water (1 : 1) at 3.2 × 10^−2^ M. Measurements were conducted at *t* = 10 min (panels a–c), 45 min (panels d–f) and 60 min (panels g–i). For each timepoint, control ^1^H MR (H_2_O signal) is displayed in left column, ^19^F signal in the center column, and overlay in the right column. ^19^F concentration (mM) is indicated next to the corresponding sample.

To investigate the potential of trifluoroborate units as ^19^F MRI probes, relaxivity studies were carried out at a 7.1 T field, on a CH_3_CN/H_2_O (1 : 1) solution of Zn(Do2py2BF_3_) at a concentration of 0.032 M in complex (*i.e.*^19^F concentration of 0.192 M owing to the 6 fluorine atoms per molecule). A single peak in ^19^F modality was detectable at −137 ppm. Longitudinal (*T*_1_) relaxivity was measured with an inversion recovery sequence, while the transversal (*T*_2_) relaxivity was measured with a CPMG sequence, leading to values of *T*_1_ = 383.7 ± 0.01 ms and *T*_2_ = 268 ± 0.02 ms. These are reasonably short relaxation times compared to corresponding ligands bearing trifluoromethyl groups (*i.e.* two to three times lower *T*_1_ than cyclam methylene-CF_3_ analogue with diamagnetic Cu(i) cation),^5,38^ that allowed a large number of averages to be accumulated in a short time. Then a phantom was prepared with variable ^19^F concentrations ranging from 1 mM to 150 mM (Fig. 2). Samples with ^19^F concentration of 50, 100 and 150 mM were clearly detectable already after 10 min of acquisition (corresponding Zn(Do2py2BF_3_) concentration of 8.3, 16.7 and 25 mM, respectively). After 45 min of acquisition, samples with 15 and 25 mM ^19^F were barely visible so a different phantom (25–150 mM) was used for detection limit measurement with 10, 45 and 60 minutes acquisition (Fig. 2). From these data, a detection limit between 50 and 75 mM in ^19^F was observed at *t* = 10 minutes, and of 40 mM in ^19^F (6.7 mM in complex) could be determined for acquisition times of 45 and 60 minutes. This value is clearly in the range of perfluorocarbons used in preclinical and clinical studies.^52^

## Conclusions

In conclusion, we have successfully prepared the first example of a small trifluoroborated macrocyclic chelator, through a practical synthetic procedure and easy purifications. This ligand presents suitable stability of the BF_3_ units, which is the usual limitation of such functions for *in vivo* applications, and a spectator character of these groups upon coordination to Zn(ii) in the corresponding model complex. This architecture subsequently allowed easy late-stage ^18^F radiolabeling through simple and fast isotopic exchange with great RCY, and trifluoroborates have also been used for the first time as ^19^F MRI reporters. These first results suggest that such BF_3_-appended azamacrocycles are a new very promising class of chelators to envisage both theranostic applications with the ^18^F PET modality and suitable radiometal coordination design, and their use as sensitive or responsive contrast agents when using the ^19^F MRI modality. Following this first step, ligand optimization will now be tackled for the various metals and radiometals relevant for these two distinct applications.

## Data availability

CCDC 2314190 (H_2_Do2py2BF_3_ ligand), 2314191 (H_4_Do2py2BF_3_^2+^ ligand) and 2314192 (Zn(Do2py2BF_3_)) contain the ESI† for this paper. The datasets supporting this article have been uploaded as part of the ESI.† Additional data can be provided upon reasonable request to Thibault.troadec@univ-brest.f.

## Author contributions

Conceptualization and funding acquisition by TT; investigation by CS, TT, VM, NSM and FG; supervision by TT, RT, LT, ET; original draft by TT; review and editing by all authors.

## Conflicts of interest

There are no conflicts to declare.

## Supplementary Material

SC-015-D4SC02871F-s001

SC-015-D4SC02871F-s002
