# Supplementary material for: A versatile fluorinated azamacrocyclic chelator enabling 18F PET or 19F MRI: a first step towards new multimodal and smart contrast agents
Source: Chem Sci. 2024 Jul 25;15(33):13550–7. doi: 10.1039/d4sc02871f (PMC11339802; doi:10.1039/d4sc02871f)
Supplement: SC-015-D4SC02871F-s001 [file SC-015-D4SC02871F-s001.pdf]

Supplementary Information (SI) for Chemical Science.
This journal is © The Royal Society of Chemistry 2024

A versatile fluorinated azamacrocyclic chelator enabling ^{18}F PET or ^{19}F MRI : a first step towards new multimodal and smart contrast agents.

Charline Sire,[a] Vincent Meneyrol,[b] Nathalie Saffon-Merceron,[c] Enzo Terreno,[d] Francesca Garello,[d] Lorenzo Tei,[e] Emmanuelle Jestin,[b] Raphaël Tripier[a] and Thibault Troadec*[a]

[a.] *Univ. Brest, UMR CNRS 6521 CEMCA, 6 Avenue Victor Le Gorgeu, 29200 Brest, France.*
E-mail: Thibault.troadec@univ-brest.fr
[b.] *Cyclotron Réunion Océan Indien CYROI, 2 rue Maxime Rivière 97490 Sainte-Clotilde, France.*
[c.] *Institut de Chimie de Toulouse (UAR 2599), 118 route de Narbonne, 31062 Toulouse Cedex 9, France.*
[d.] *Department of Molecular Biotechnology and Health Sciences, University of Turin, Piazza Nizza 44/bis, 10126 Turin (Italy).*
[e.] *Dipartimento di Scienze ed Innovazione Tecnologica, Università del Piemonte Orientale "Amedeo Avogadro", Viale T. Michel 11, 15121 Alessandria, Italy.*

Corresponding author e-mail: Thibault.troadec@univ-brest.fr

Electronic Supporting Information

General considerations......2
Synthesis and characterization of H_2Do2py2BF_3 :......3
Figure S1 - ^{1}H NMR (400.16 MHz) of Do2py2BPin intermediate......3
Figure S2 - ^{1}H NMR (400.16 MHz) of H_2Do2py2BF_3......5
Figure S3 - ^{19}F NMR (376.53 MHz) of H_2Do2py2BF_3......6
Figure S4 - ^{11}B NMR (128.39 MHz) of H_2Do2py2BF_3......7
Figure S5 – ^{13}C (JMod) NMR (125.79 MHz) of H_2Do2py2BF_3 at pH 4 (D_2O/CD_3CN 1:1)......7
Figure S6 - ^{11}B-^{1}H HMBC NMR of H_2Do2py2BF_3......8
Figure S7 - COSY NMR of H_2Do2py2BF_3......8
Figure S8 – ^{13}C-^{1}H HSQC NMR of H_2Do2py2BF_3......9
Figure S9 - ESI-HRMS (Positive ions) of H_2Do2py2BF_3......10
Figure S10 - ESI-HRMS (Negative ions) of H_2Do2py2BF_3......11
Synthesis and characterization of Zn(Do2py2BF_3)......12
Figure S11 - ^{1}H NMR (400.16 MHz, D_2O/CD_3CN 1 :1) of Zn(Do2py2BF_3) at 298K and 333K......13
Figure S12 - ^{19}F NMR (376.53 MHz, D_2O/CD_3CN 1 :1) of Zn(Do2py2BF_3) at 298K......14
Figure S13 – X-Ray diffraction structure of Zn(Do2py2BF_3) with calculated N1-N2-N3-N4 centroid and Zn-N distances......15
Figure S14 - ESI-HRMS (Positive ions) of Zn(Do2py2BF_3)......16
X-Ray diffraction data:......17
H_2Do2py2BF_3 (pH 4)......17
Table S1 - Crystal data and structure refinement for H_2Do2py2BF_3 (pH 4)......18
H_4Do2py2BF_3^{2+} (pH 1)......19
Table S2 - Crystal data and structure refinement for H_4Do2py2BF_3^{2+} (pH 1)......20

Zn(Do2py2BF$_3$) complex....21
Table S3 - Crystal data and structure refinement for Zn(Do2py2BF$_3$)....22
NMR monitoring of Do2py2BF$_3$ and Zn(Do2py2BF$_3$) pH-dependant stability....23
Figure S15: H$_2$Do2py2BF$_3$ ligand (pH 2, 25°C)....23
Figure S16: H$_2$Do2py2BF$_3$ ligand (pH 7.3)....24
Figure S17: H$_2$Do2py2BF$_3$ ligand (pH 9.7)....24
Figure S18: Zn(Do2py2BF$_3$) complex (pH 2)....25
Figure S19: Zn(Do2py2BF$_3$) complex (pH 7.3)....26
Figure S20: Zn(Do2py2BF$_3$) complex (pH 9.7)....27
^{18}F-Radiolabeling....28
Figure S21: Scheme of TRACERLab FX FN module.....29
Figure S22: HPLC analysis of H$_2$Do2Py2BF$_3$ radiolabeling.....30
Figure S23: HPLC analysis of Zn(Do2Py2BF$_3$) radiolabeling.....32
^{19}F-Magnetic Resonance....34
Figure S24: ^{19}F Signal Intensity values at 7T....34
References....34

General considerations

Reagents were purchased from Sigma-Aldrich, TCI Chemicals, Acros Organics. Dipyridylcyclen (Do2py) was prepared according to previously described procedure.[1]
NMR data were recorded at the "Service commun de RMN-RPE" at the Université de Bretagne Occidentale (UBO). ^{1}H, ^{13}C and 2D NMR spectra were recorded on Bruker Avance III HD 500 (500.13 MHz for ^{1}H, 125.79 MHz for ^{13}C, 470.59 MHz for ^{19}F and 202.46 for ^{11}B), Bruker Avance 400 or 400 Neo (400.13 MHz for ^{1}H,100.61 MHz for ^{13}C, 376.50 MHz for ^{19}F and 161.98 for ^{11}B) or Bruker AMX-3 300 (300.13 MHz for ^{1}H, 75.47 MHz for ^{13}C and 282.40 MHz for ^{19}F) spectrometers. All ^{13}C, ^{11}B and ^{19}F spectra are proton-decoupled unless otherwise stated. Signal multiplicity are reported as follows: d = doublet, dd = doublet of doublet, t = triplet, q = quadruplet, br = broad.
High-resolution mass spectra (HRMS) were performed on a Bruker maXis mass spectrometer by the SALSA platform from ICOA laboratory (Orléans, France). Elemental analysis was conducted at CRMPO platform (ISCR, Rennes) with reported average values of 2 experiments.

Crystallographic data collection was performed at low temperature (193 K) at the Service RX of the Institut de Chimie de Toulouse, Université Paul Sabatier, on a Bruker-AXS D8-Venture diffractometer using MoKα radiation (λ = 0.71073 Å) for H$_2$**Do2py2BF3** and **Zn(Do2py2BF3)** or CuKα radiation (λ = 1.54178 Å) for H$_4$**Do2py2BF3^{2+}**. Phi- and omega-scans were used. The data were integrated with SAINT (Program for data reduction, Bruker-AXS) and an empirical absorption correction with SADABS was applied (Program for data correction, Bruker-AXS). The structures were solved using an intrinsic phasing method (ShelXT)55 and refined using a least-squares method on F2.56 All non-H atoms were refined with anisotropic displacement parameters. Hydrogen atoms were refined isotropically at calculed positions using a riding model except H atoms on nitrogen and water molecules (located by difference

Fourier maps). Some parts of the H_2**Do2py2BF3** ligand and a water molecule of the **Zn(Do2py2BF3)** complex were found to be disordered. Several restraints (SAME, SIMU, DELU) were applied to refine some moieties of the molecules and to avoid the collapse of the structures during the least-squares refinement by the large anisotropic displacement parameters.

CCDC 2314190 (H_2**Do2py2BF$_3$ ligand**), 2314191 (H_4**Do2py2BF$_3^{2+}$ ligand**) and 2314192 (**Zn(Do2py2BF$_3$)**) contain the supplementary crystallographic data for this paper. These data can be obtained free of charge from The Cambridge Crystallographic Data Centre via https://www.ccdc.cam.ac.uk/structures/.

Synthesis and characterization of H_2Do2py2BF$_3$:

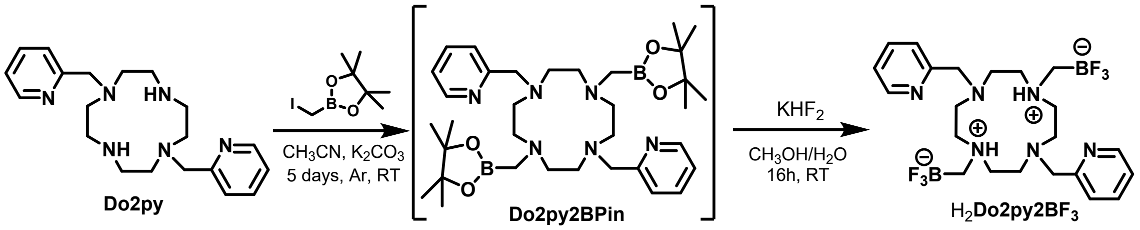

Warning: potassium bifluoride (KHF_2) is abrasive for glassware.
Under argon, Do2py (0.500 g, 1.40 mmol) was placed in dry degassed acetonitrile (30 mL) with potassium carbonate (1.0 g, 5 equiv.) and a solution of iodomethylpinacolborane (0.788 g, 3.08 mmol, 2.2 equiv.) in dry acetonitrile (20 mL) was added dropwise. The mixture was stirred at RT for 3 days, then filtered to remove excess potassium carbonate and the volatiles were removed under vaccum to obtain crude **Do2py2BPin**.

Do2py2BPin : ^{1}H NMR (CD_3CN, 400.13 MHz, 298 K, TMS) δ (ppm) 8.63 (d, 2H, $J_{H\text{-}H}$ = 4.8 Hz CH_{Ar}), 7.79 (dt, 2H, $J_{H\text{-}H}$ = 7.7 and 1.9 Hz, CH_{Ar}), 7.34 (brt, 4H, $J_{H\text{-}H}$ = 7.7 Hz, CH_{Ar}), 4.22 (brs, <4H, CH_2Py), 3.40-2.45 (br, <16H, CH_2^{cyclen}), 1.45-1.05 (br, 28H, 2 x CH_2BPin + 8 x CH_3^{Pin})

re S1 - ^{1}H NMR (400.16 MHz) of Do2py2BPin intermediate

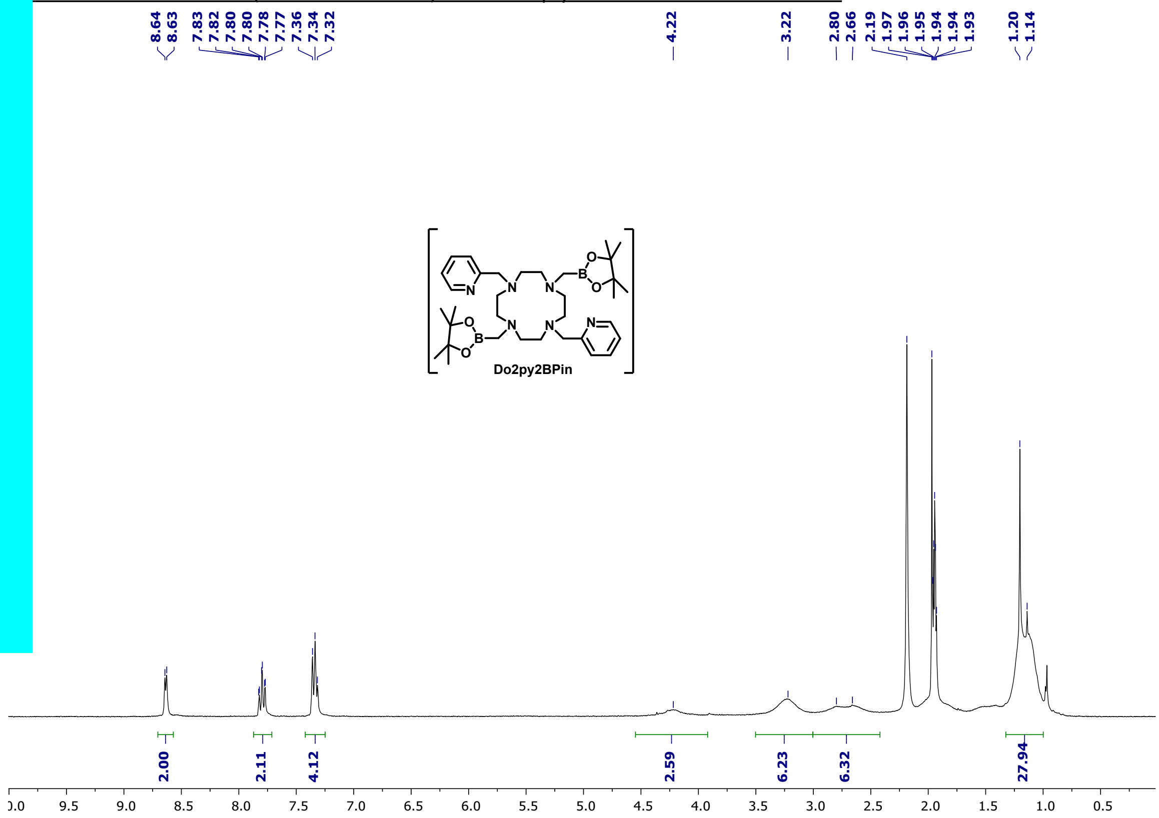

Crude **Do2py2BPin** was redissolved in methanol (16 mL) and potassium bifluoride (1.1 g, 14 mmol, 10 equiv.) in water (4 mL) was added. The mixture was stirred at RT for 16h, then volatiles were removed under vaccum. The product was extract with acetonitrile (2 x 10 mL), dried under vacuum, then washed with sodium thiosulfate aqueous solution (2M) (2 x 5 mL) to provide H_2Do2py2BF_3 as a white powder (444 mg, 0.86 mmol, yield = 61%).

H_2Do2py2BF_3 *(pH 4)*:

^{1}<u>H NMR</u> (D_2O/CD_3CN 1:1, 400.13 MHz, 298 K, TMS) δ (ppm): 8.53 (d, 2H, J_{H-H} = 4.9 Hz CH_{Ar}), 7.78 (dt, 2H, J_{H-H} = 7.7 and 1.8 Hz, CH_{Ar}), 7.48 (d, 2H, J_{H-H} = 7.7 Hz, CH_{Ar}), 7.33 (dd, 2H, J_{H-H} = 7.7 and 4.9 Hz, CH_{Ar}), 3.92 (s, 4H, CH_2Py), 3.28 (m, 8H, CH_2^{cyclen}), 2.91 (m, 8H, CH_2^{cyclen}), 1.80 (q, 4H, $^{3}J_{H-F}$ = 5.2 Hz, CH_2BF_3).

^{19}<u>F NMR</u> (D_2O/CD_3CN 1:1, 376.50 MHz, 298 K, $CFCl_3$) δ (ppm): -135.6 (s).

^{11}<u>B NMR</u> (D_2O/CD_3CN 1:1, 161.98 MHz, 298 K, BF3·Et2O) δ (ppm): 2.4 (s).

^{13}<u>C Jmod NMR</u> (D_2O/CD_3CN 1:1, 125.79 MHz, 298 K, TMS) δ (ppm): 156.8 (C^{Ar}), 149.2 (CH^{Ar}), 136.7 (CH^{Ar}), 123.1 (CH^{Ar}), 122.2 (CH^{Ar}), 57.2 (CH_2Py), 51.7 (CH_2^{cyclen}), 48.4 (CH_2^{cyclen}), 47.8 (CH_2BF_3).

<u>ESI-HR-MS (positive ions, M = H_2Do2py2BF_3)</u>:

m/z calcd. for $[C22H35B2F6N6]^{+}$: 519.3008, found $[M+H]^{+}$: 519.3012.

m/z calcd. for $[C22H36B2F6N6]^{2+}$: 260.1540, found $[M+2H]^{2+}$: 260.1542.

additional fragmentations found: $[MH_2\text{-}HF]^{2+}$: m/z 250.1510, $[MH_2\text{-}2HF]^{2+}$: m/z 240.1479.

<u>ESI-HR-MS (negative ions, M = H_2Do2py2BF_3)</u>:

m/z calcd. for $[C22H33B2F6N6]^{-}$: 517.2862, found $[M-H]^{-}$: 517.2864.

m/z calcd. for $[C22H32B2F6N6Na]^{-}$: 539.2682, found $[M-2H+Na]^{-}$: 539.2684.

m/z calcd. for $[C22H34B2F6N6I]^{-}$: 645.1985, found $[M+I]^{-}$: 685.1986.

<u>Elemental analysis:</u>

calcd (%) for C22H34B2F6N6: C 51.00, H 6.61, N 16.22; found: C 50.67, H 6.59, N 16.22

H_2Do2py2BF_3 *(pH 1)*:

^{1}H NMR (D_2O, 400.13 MHz, 298 K, TMS) *δ (ppm):* 8.83 (d, 2H, J_{H-H} = 6.1 Hz CH_{Ar}), 8.63 (m, 2H, CH_{Ar}), 8.27-8.14 (m, 2H, CH_{Ar}), 8.05 (m, 2H, CH_{Ar}), 4.18 (m, 4H, CH_2Py), 3.53 (br, 8H, CH_2^{cyclen}), 3.09 (br, 4H, CH_2^{cyclen}), 2.93 (br, 4H, CH_2^{cyclen}), 2.30-1.90 (brm, 4H, CH_2BF_3).
^{19}F NMR (D_2O/CD_3CN 1:1, 376.50 MHz, 298 K, $CFCl_3$) *δ (ppm):* -136/-139 ppm (m).
^{11}B NMR (D_2O/CD_3CN 1:1, 161.98 MHz, 298 K, $BF3·Et2O$) *δ (ppm):* 2.1 (s).

Figure S2 - ^{1}H NMR (400.16 MHz) of $H_2Do2py2BF_3$

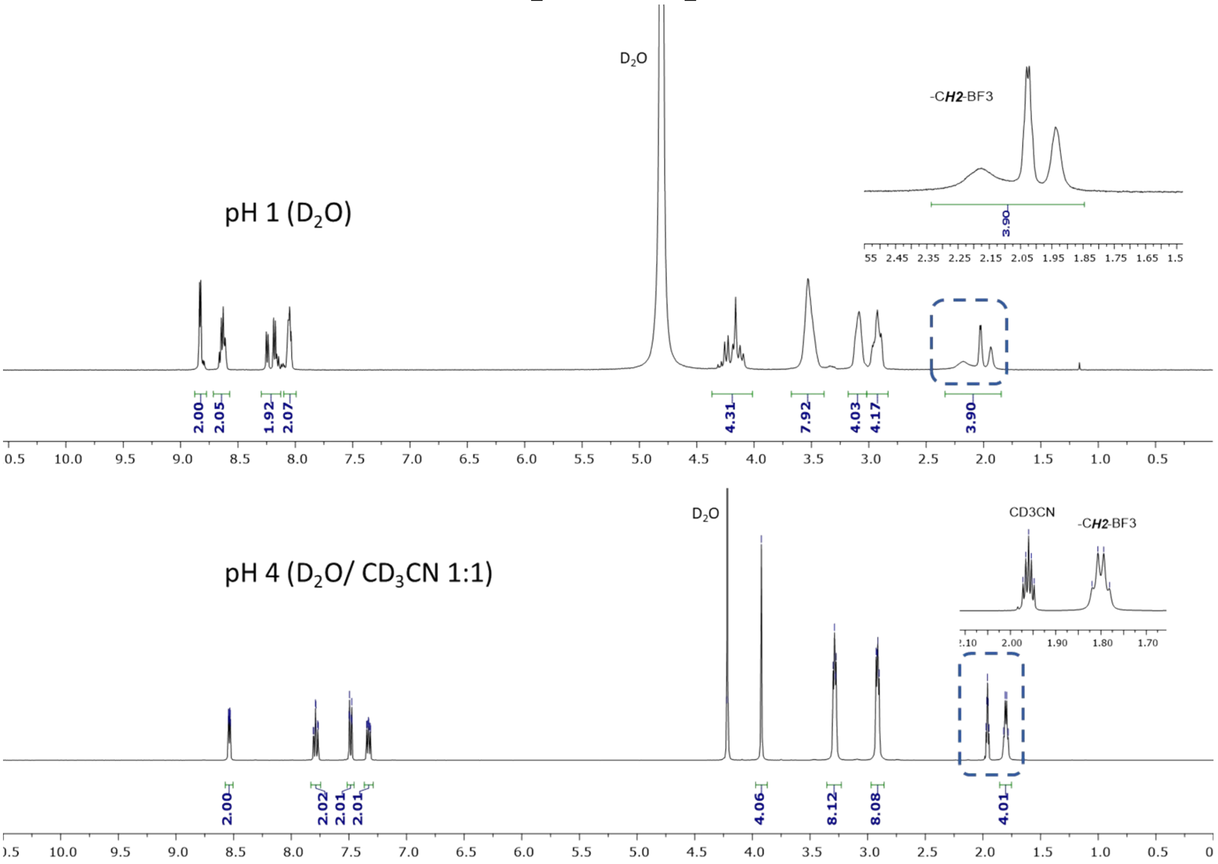

Figure S3 - ^{19}F NMR (376.53 MHz) of H_2Do2py2BF_3

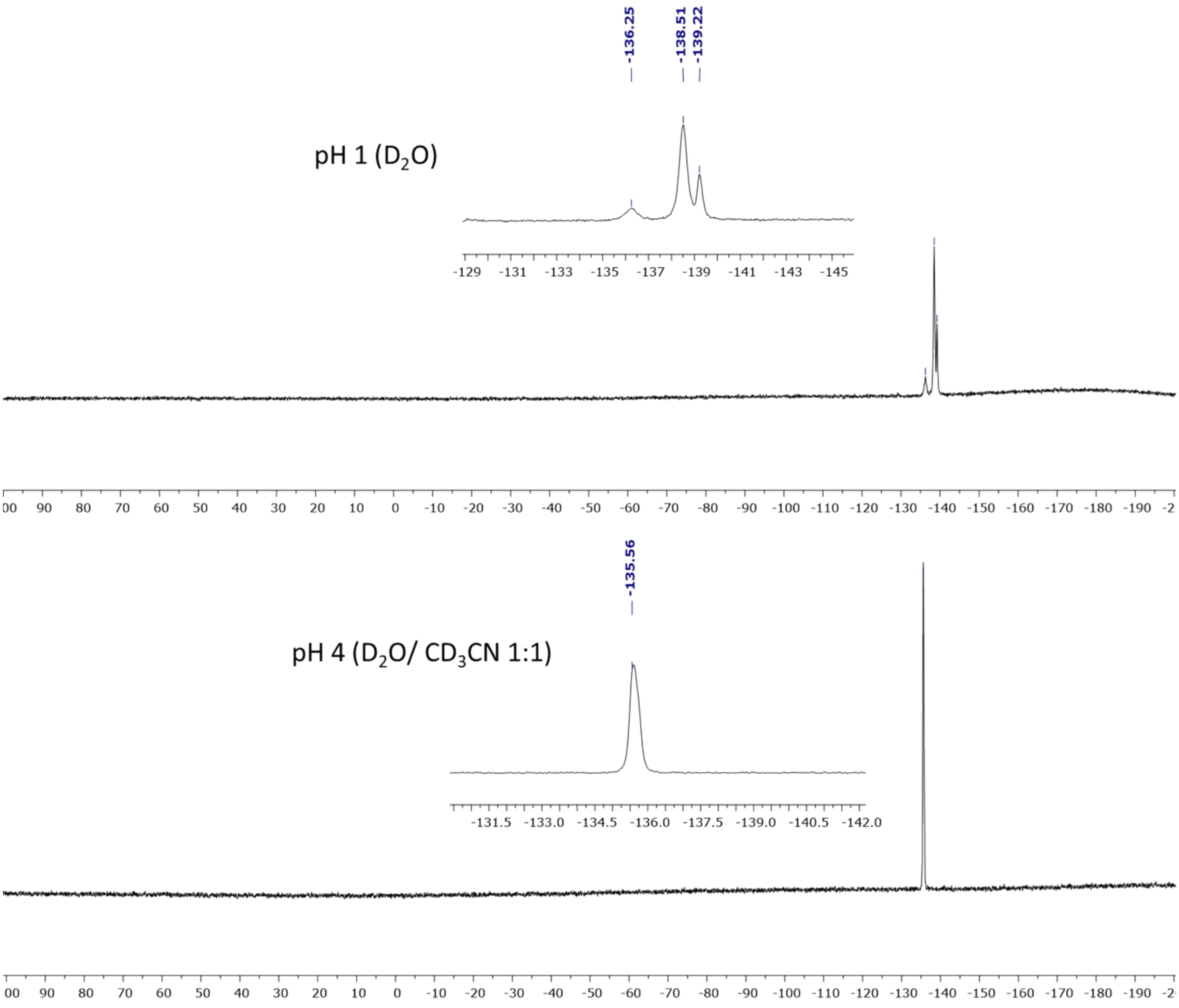

Figure S4 - ^{11}B NMR (128.39 MHz) of $H_2Do2py2BF_3$

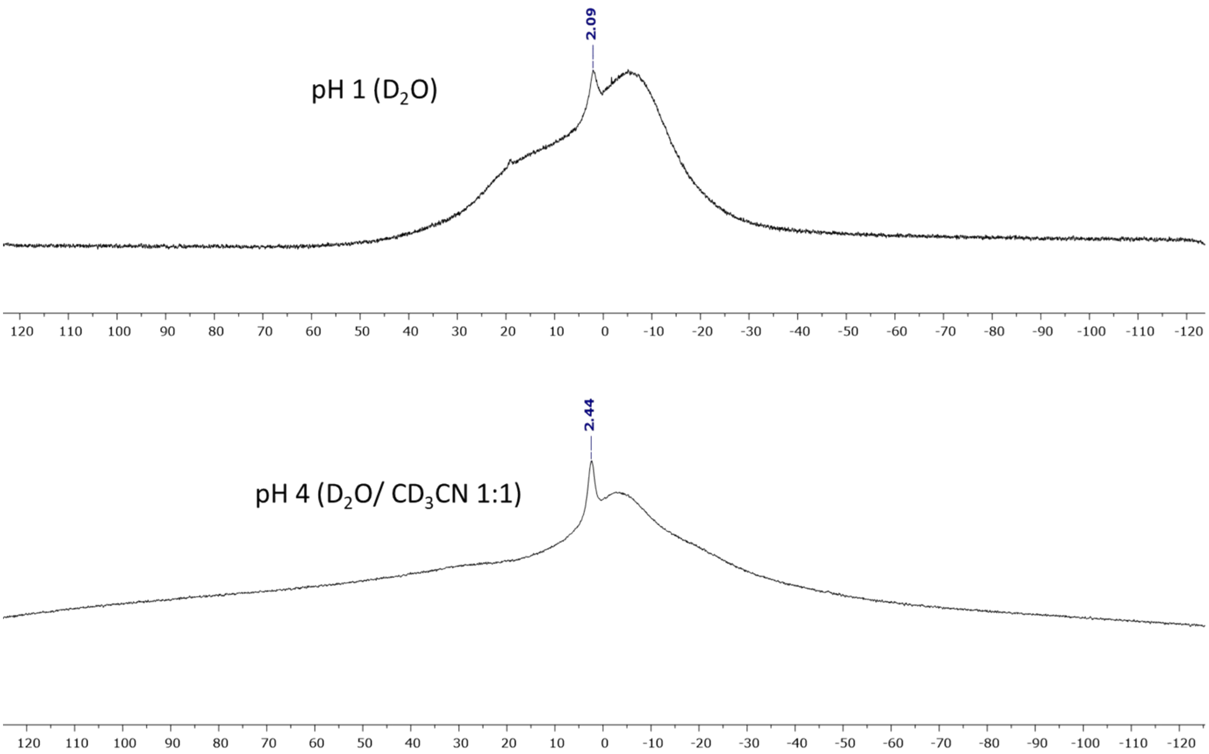

Figure S5 – ^{13}C (JMod) NMR (125.79 MHz) of $H_2Do2py2BF_3$ at pH 4 (D_2O/CD_3CN 1:1)

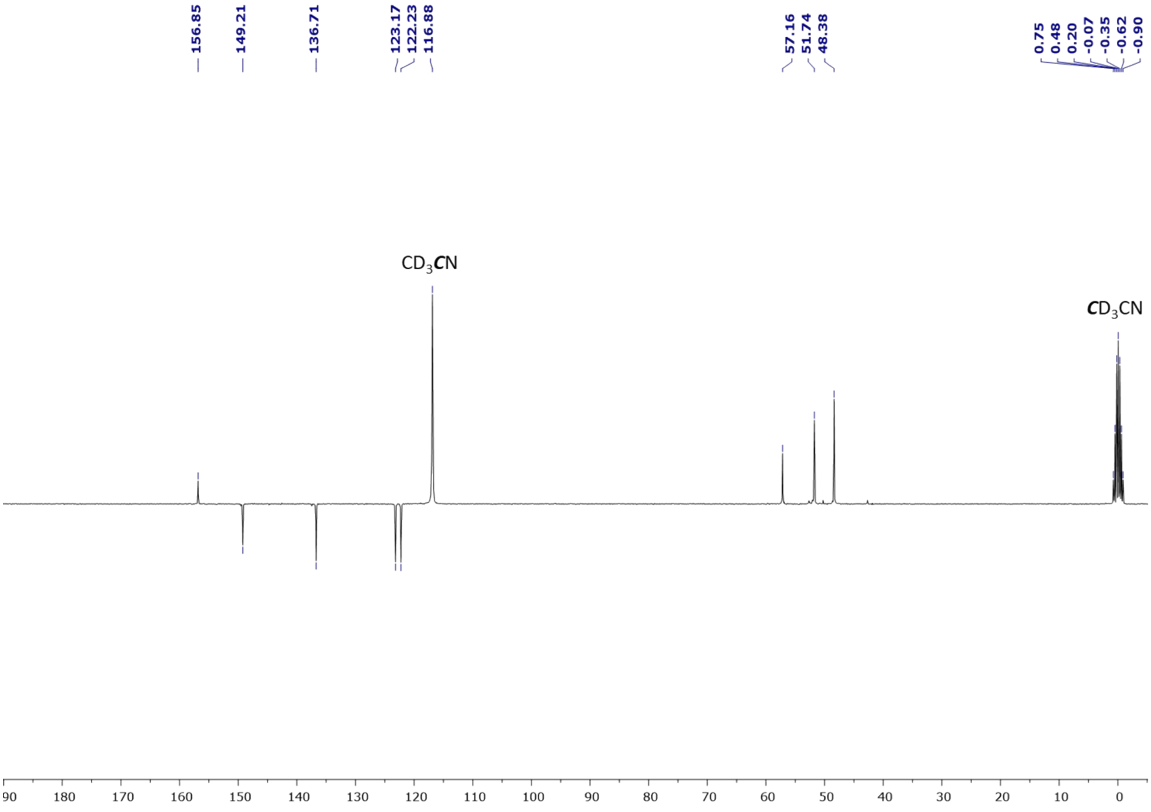

Figure S6 - ^{11}B-^{1}H HMBC NMR of $H_2Do2py2BF_3$

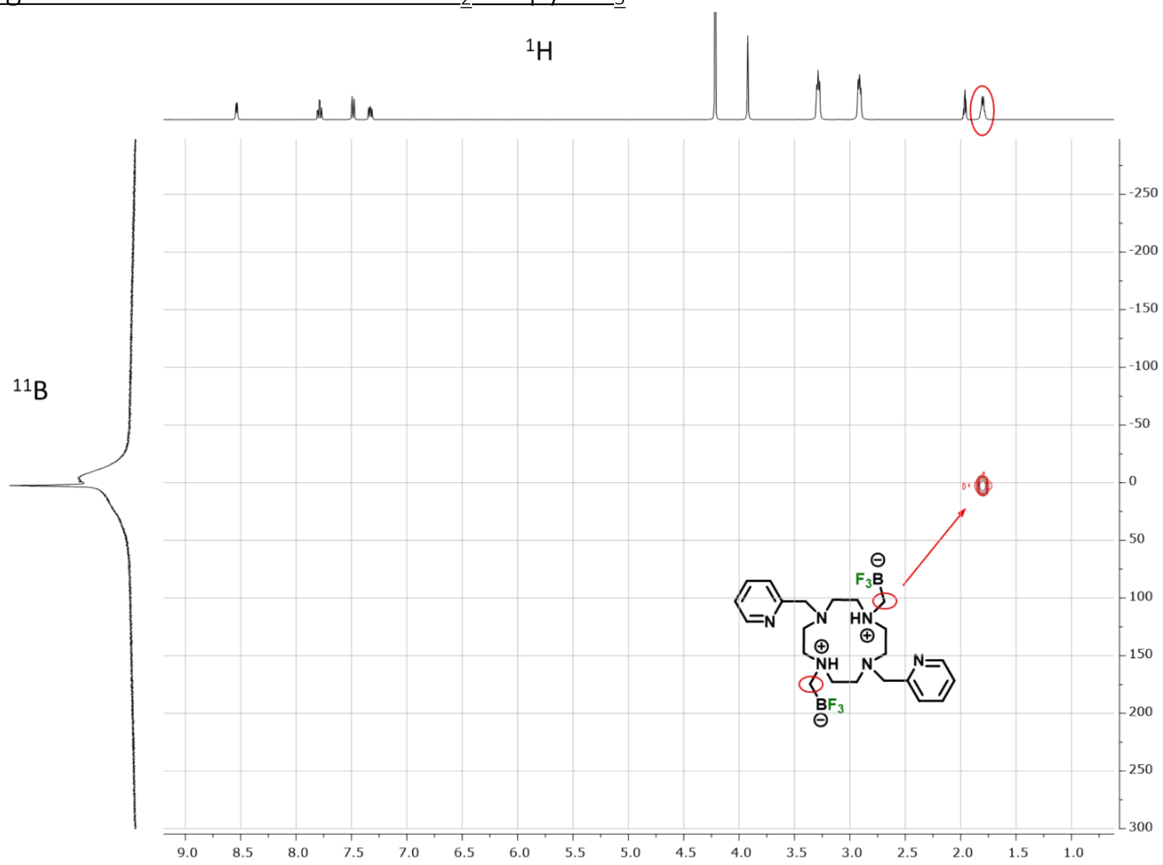

Figure S7 - COSY NMR of $H_2Do2py2BF_3$

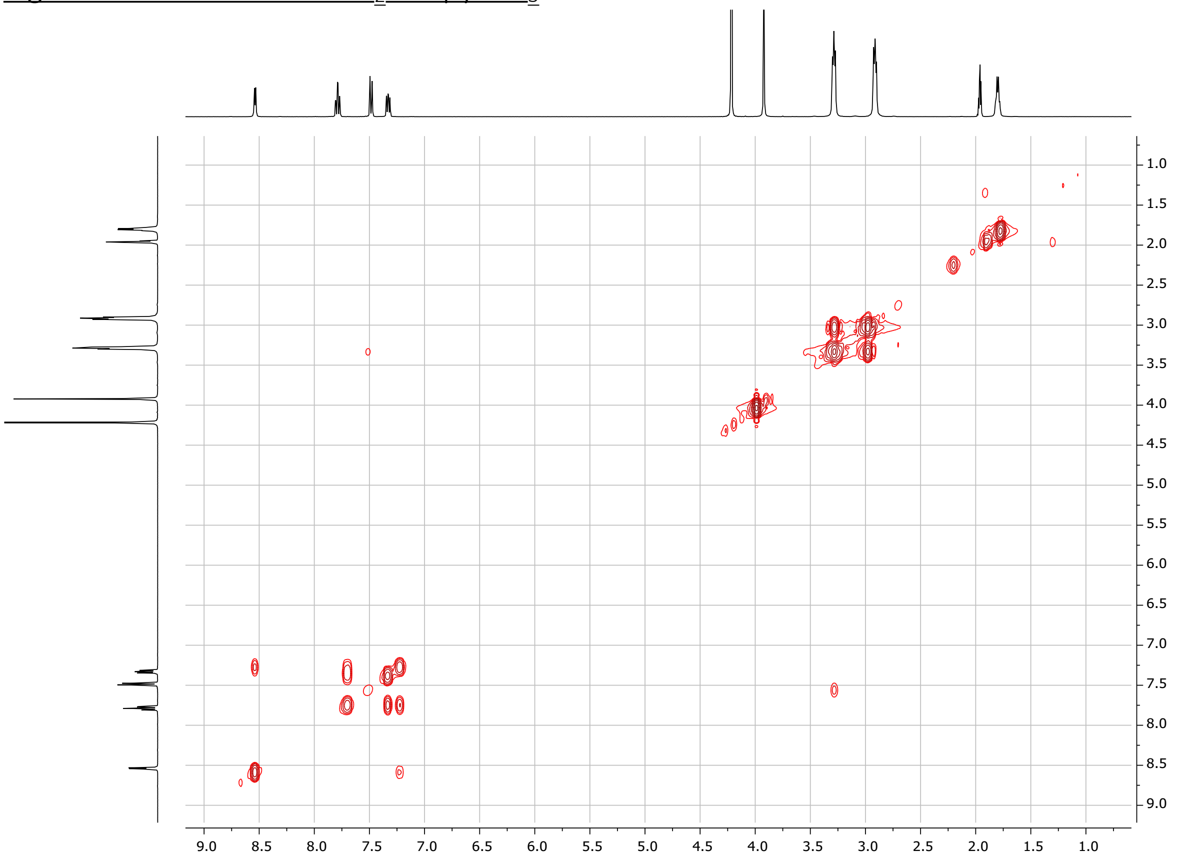

Figure S8 – ^{13}C-^{1}H HSQC NMR of H_2Do2py2BF_3

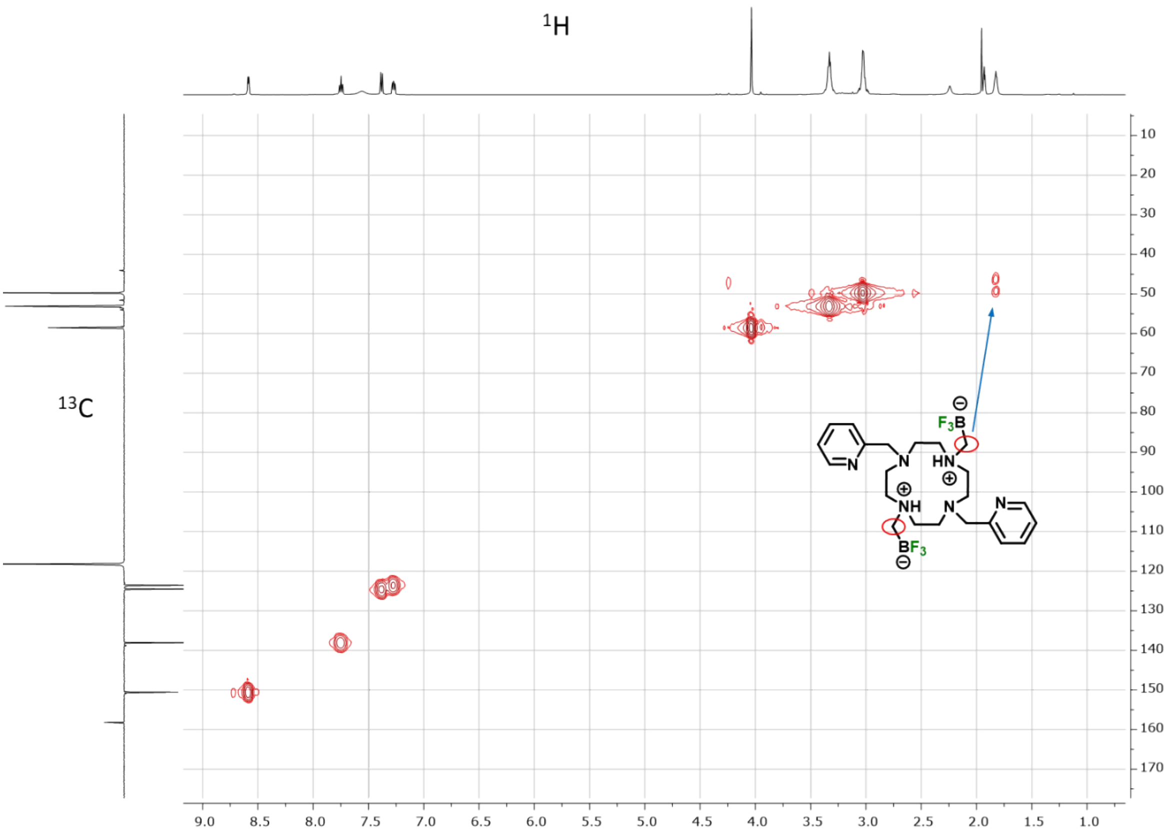

Figure S9 - ESI-HRMS (Positive ions) of $H_2Do2py2BF_3$

Fédération de Recherche Physique et Chimie du Vivant (FR2708 : CBM/ICOA)
Plate-forme de Spectrométrie de Masse Haute Résolution

HRAM

Analysis Info

Sample Name	**Do2py2BF3**	Acquisition Date	01/04/2019 14:08:22
Analysis Name	X049086CYC.d	Instrument / Ser#	maXis 255552.00086
		Method	positif-6.m

Acquisition Parameter

Source Type	ESI	Ion Polarity	Positive	Set Nebulizer	0.6 Bar
Scan Begin	50 m/z	Set Capillary	4500 V	Set Dry Heater	200 °C
Scan End	2500 m/z	Set Collision Cell RF	1800.0 Vpp	Set Dry Gas	7.0 l/min

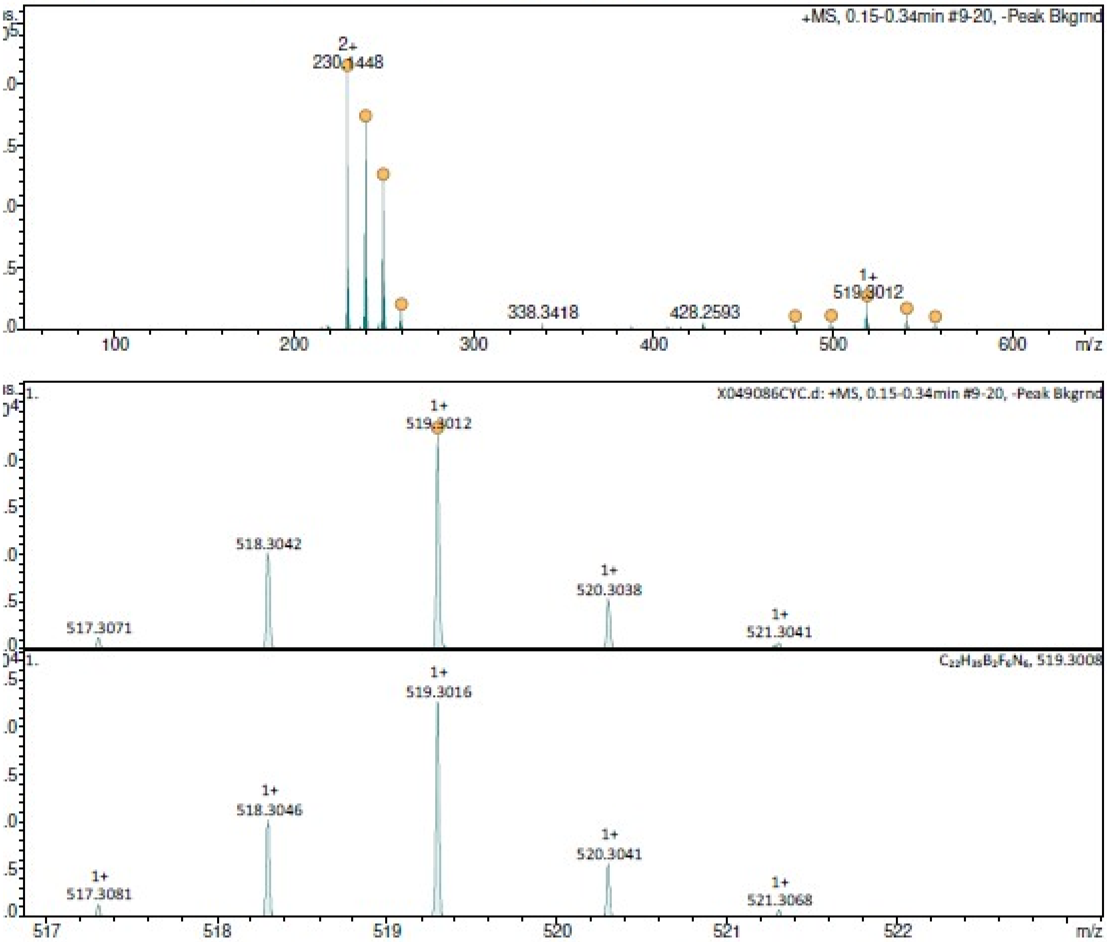

Meas. m/z	z	#	Ion Formula	m/z	err [ppm]	mSigma	rdb	e⁻ Conf
230.144838	2+	1	C22H33B2F3N6	230.144697	1.1	6.6	10.0	even
	2+	2	C24H36B2F6	230.144817	1.7	6.6	6.0	even
240.147949	2+	1	C22H34B2F4N6	240.147811	1.0	6.9	9.0	even
250.151078	2+	1	C22H35B2F5N6	250.150925	0.9	11.5	8.0	even
260.154179	2+	1	C22H36B2F6N6	260.154039	1.0	15.2	7.0	even
479.288318	1+	1	C22H33B2F4N6	479.288345	1.7	10.4	9.0	even
499.294582	1+	1	C22H34B2F5N6	499.294573	1.5	20.2	8.0	even
519.301240	1+	1	C22H35B2F6N6	519.300802	0.7	7.1	7.0	even

Institut de Chimie Organique et Analytique
UMR 7311 - Université d'Orléans - CNRS
BP 6759 - rue de Chartres - F-45067 ORLEANS Cedex 2
cyril.colas@univ-orleans.fr
+33 (0)2 38 49 46 61
printed: 12/04/2019 12:42:48
Page 1 of 2

Figure S10 - ESI-HRMS (Negative ions) of H_2Do2py2BF_3

Fédération de Recherche Physique et Chimie du Vivant (FR2708 : CBM/ICOA)
Plate-forme de Spectrométrie de Masse Haute Résolution

HRAM

Analysis Info

Sample Name	Do2py2BF3	Acquisition Date	01/04/2019 20:53:55
Analysis Name	X049087CYC.d	Instrument / Ser#	maXis 255552.00086
		Method	negatif-6.m

Acquisition Parameter

Source Type	ESI	Ion Polarity	Negative	Set Nebulizer	0.6 Bar
Scan Begin	50 m/z	Set Capillary	4000 V	Set Dry Heater	200 °C
Scan End	3000 m/z	Set Collision Cell RF	500.0 Vpp	Set Dry Gas	7.0 l/min

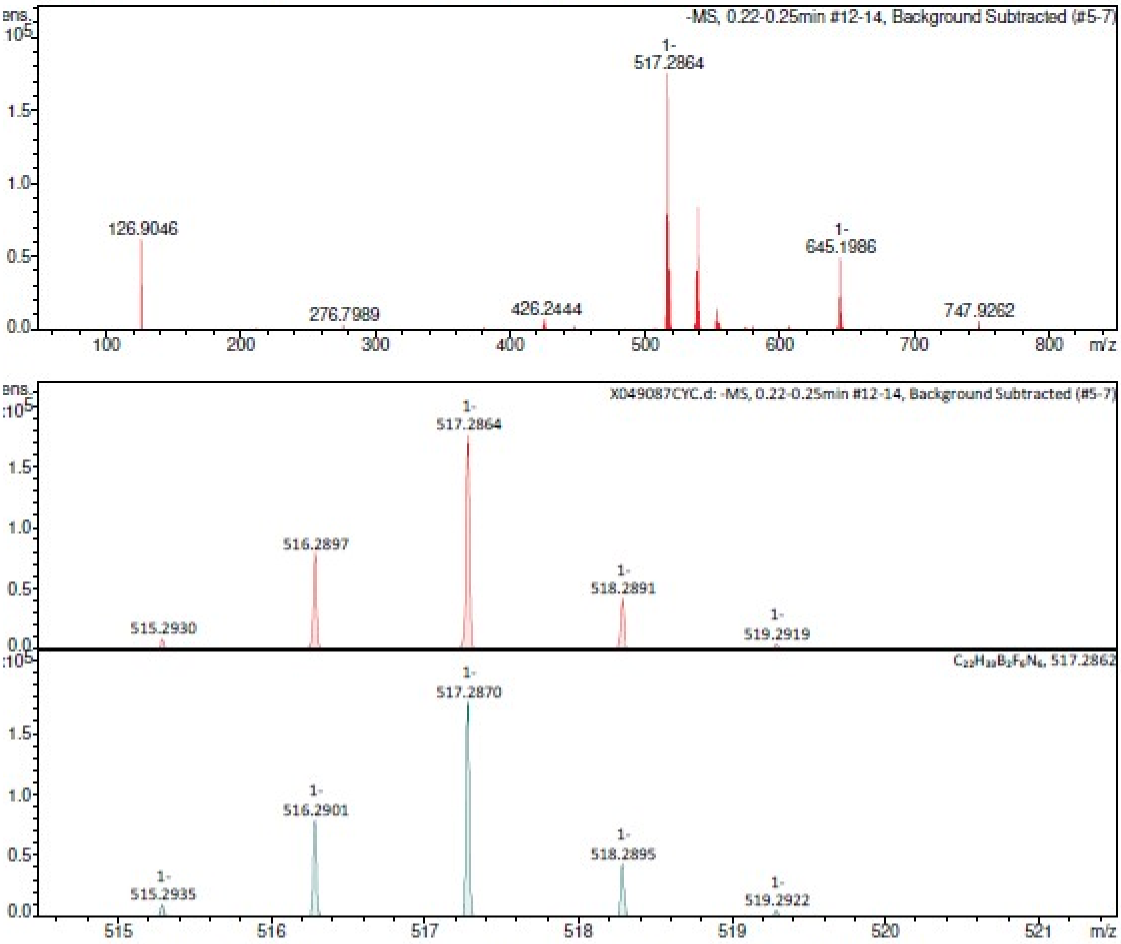

Meas. m/z	#	Ion Formula	m/z	err [ppm]	mSigma	rdb	e⁻ Conf	N-Rule
126.904556	1	I	126.905022	3.7	n.a.	0.0	even	ok
517.286441	1	C22H33B2F6N6	517.286249	1.1	5.3	7.0	even	ok
539.268437	1	C22H32B2F6N6Na	539.268193	1.0	13.7	7.0	even	ok
553.263282	1	C22H34B2ClF6N6	553.262926	0.9	7.1	6.0	even	ok
645.198624	1	C22H34B2F6IN6	645.198547	1.1	2.1	6.0	even	ok

Synthesis and characterization of Zn(Do2py2BF$_3$)

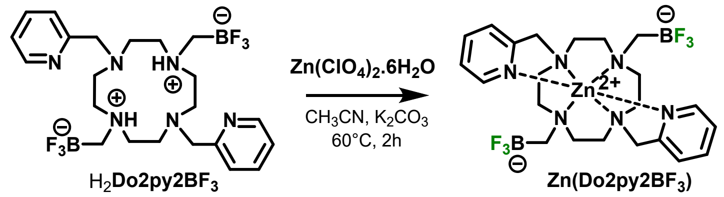

Warning: perchlorate salts can be explosive, and were therefore used in small quantities (< 100 mg) under their hydrated form.

H$_2$Do2py2BF$_3$ (0.091 g, 0.176 mmol) was placed in dry acetonitrile (7 mL) with potassium carbonate (0.122 g, 5 equiv.) and zinc(II) perchlorate hexahydrate (0.0655 g, 0.176 mmol). The mixture was stirred at 60°C for 2 hours, then filtered to remove excess potassium carbonate. 3 mL of water were added to the supernatant and the solution was slowly evaporated to afford colorless crystals of the title compound (0.077 g, 0.132 mmol, yield = 75%).

Zn(Do2py2BF$_3$):

Nb : ^{1}H NMR presents broad signals at Room Temperature, therefore spectrum integration was performed at 333K. ^{11}B and ^{13}C remain broad even at 333K and did not lead to satisfactory spectra.

^{1}H NMR (D$_2$O/CD$_3$CN 1:1, 500.13 MHz, 298 K, TMS) δ (ppm): 8.42 (d, 2H, $J_{H\text{-}H}$ = 5.1 Hz CH$_{Ar}$), 7.98 (dt, 2H, $J_{H\text{-}H}$ = 7.6 and 1.5 Hz, CH$_{Ar}$), 7.50 (d, 2H, $J_{H\text{-}H}$ = 7.6 Hz, CH$_{Ar}$), 7.33 (dd, 2H, $J_{H\text{-}H}$ = 7.6 and 5.1 Hz, CH$_{Ar}$), 4.20 (br, 4H, CH$_2$Py), 3.15-2.60 (m, 16H, CH$_2^{cyclen}$), 1.13 (br, 4H, $^{3}J_{H\text{-}F}$ = 5.2 Hz, CH$_2$BF$_3$).

^{19}F NMR (D$_2$O/CD$_3$CN 1:1, 470.59 MHz, 298 K, CFCl$_3$) δ (ppm): -137.4 (s).

ESI-HR-MS (positive ions, M = Zn(Do2py2BF$_3$)):

m/z calcd. for [C22H33B2F5N6Zn]$^+$: 561.2080, found [M-F]$^+$: 561.2082.

m/z calcd. for [C22H33B2F6N6Zn]$^+$: 581.2142, found [M+H]$^+$: 581.2144.

m/z calcd. for [C22H32B2F6N6NaZn]$^+$: 603.1962, found [M+Na]$^+$: 603.1963.

m/z calcd. for [C22H32B2F6KN6Zn]$^+$: 619.1701, found [M+K]$^+$: 619.1708.

additional fragmentations found: [MH$_2$-HF]$^{2+}$: m/z 250.1510, [MH$_2$-2HF]$^{2+}$: m/z 240.1479.

Elemental analysis:

calcd (%) for C22H32B2F6N6Zn: C 45.44, H 5.55, N 14.45; found: C 45.27, H 5.49, N 15.38

Figure S11 - ^{1}H NMR (400.16 MHz, D_2O/CD_3CN 1 :1) of Zn(Do2py2BF_3) at 298K and 333K

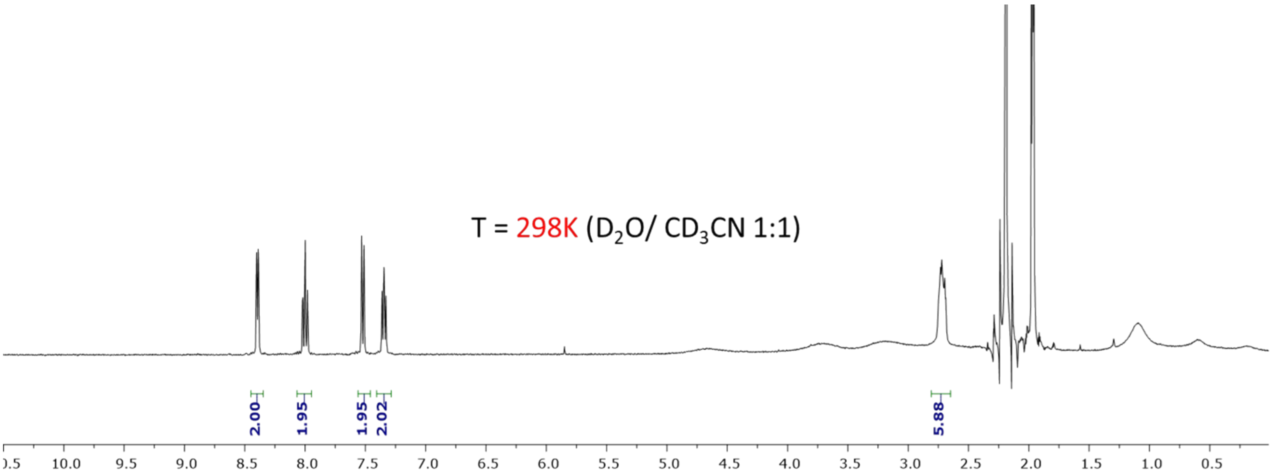

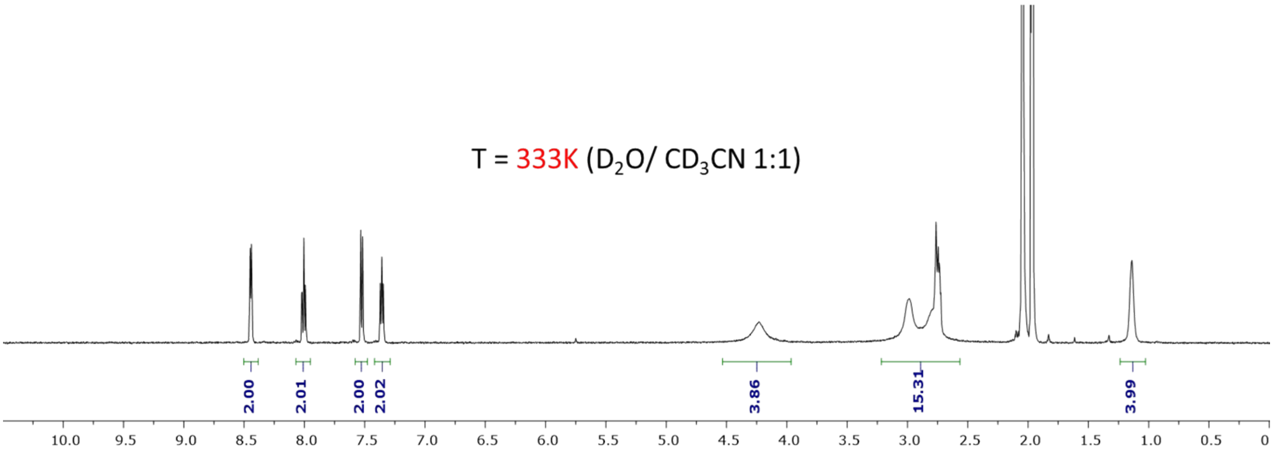

Figure S12 - ^{19}F NMR (376.53 MHz, D_2O/CD_3CN 1 :1) of Zn(Do2py2BF_3) at 298K

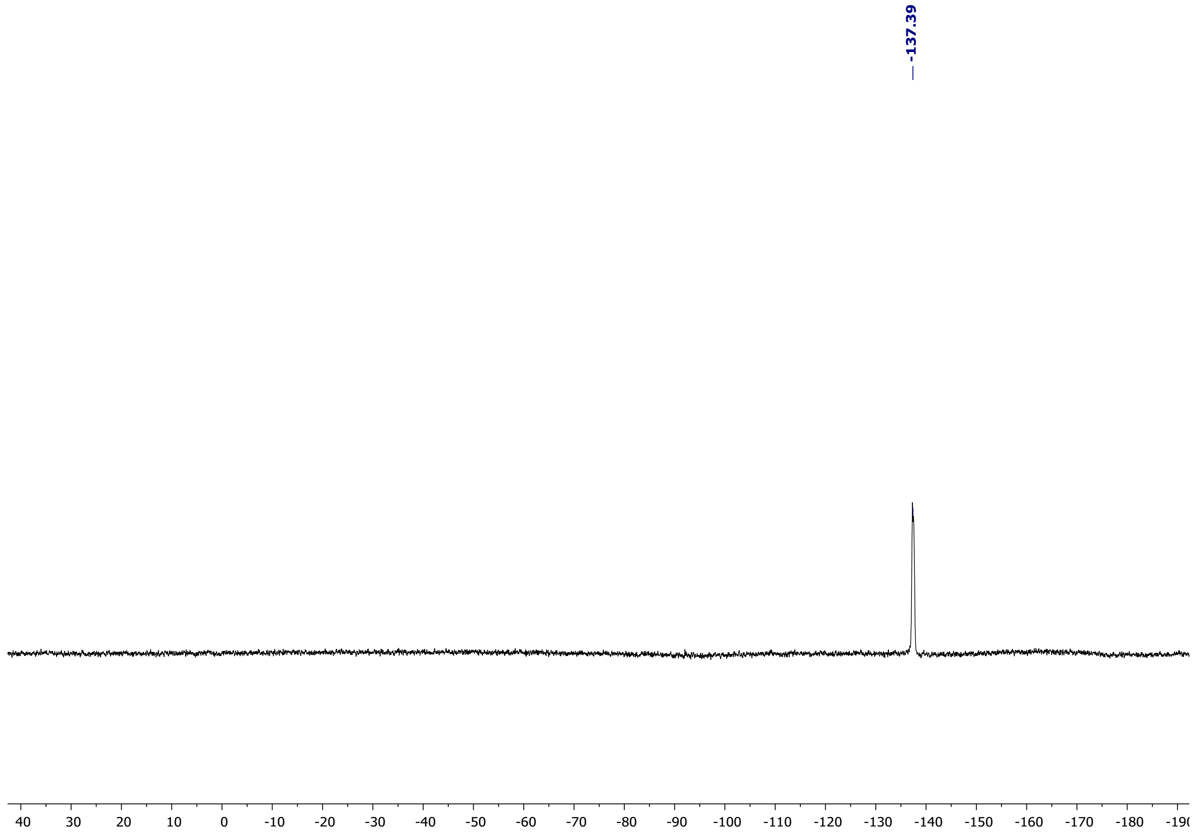

Figure S13 – X-Ray diffraction structure of Zn(Do2py2BF$_3$) with calculated N1-N2-N3-N4 centroid and Zn-N distances

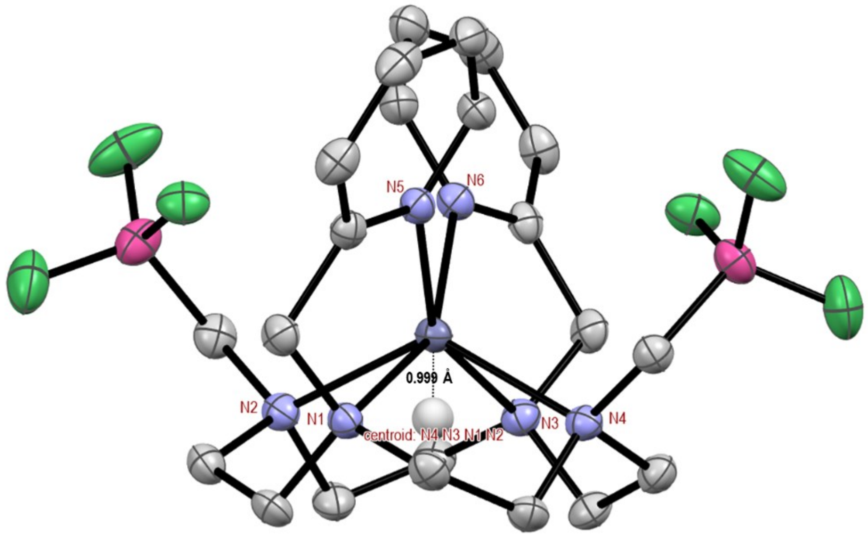

	Zn(Do2py2BF3)	Zn(Do2py)2+ (ref 2)
**Zn-N1**	2.221	2.141
**Zn-N2**	2.322	2.293
**Zn-N3**	2.244	2.172
**Zn-N4**	2.331	2.298
**Zn-N5(py)**	2.182	2.186
**Zn-N6(py)**	2.17	2.165

Bond distances (Å). Values for Zn(Do2py)$^{2+}$ from Bernier et. al [2]

Figure S14 - ESI-HRMS (Positive ions) of Zn(Do2py2BF$_3$)

Fédération de Recherche Physique et Chimie du Vivant (FR2708 : CBM/ICOA)
Plate-forme de Spectrométrie de Masse Haute Résolution

HRAM

**Analysis Info**		Acquisition Date	01/04/2019 14:09:57
Sample Name	**Do2py2BF3-Zn**	Instrument / Ser#	maXis 255552.00086
Analysis Name	X049088CYC.d	Method	positif-6.m

Acquisition Parameter

Source Type	ESI	Ion Polarity	Positive	Set Nebulizer	0.6 Bar
Scan Begin	50 m/z	Set Capillary	4500 V	Set Dry Heater	200 °C
Scan End	2500 m/z	Set Collision Cell RF	1800.0 Vpp	Set Dry Gas	7.0 l/min

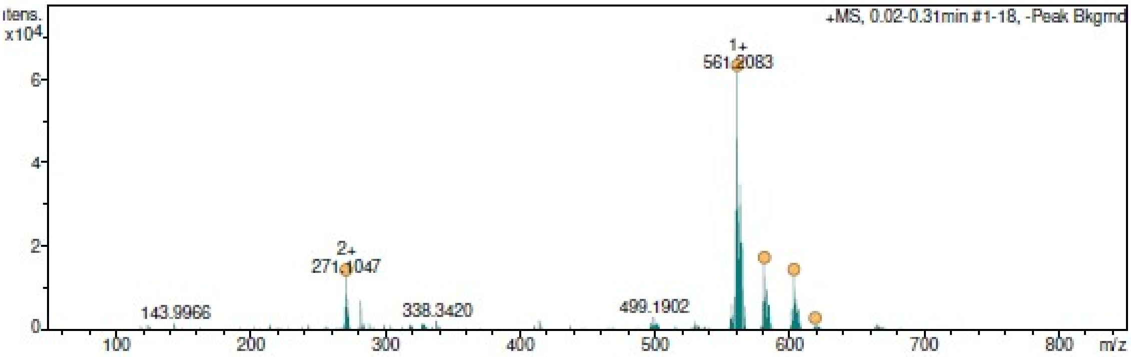

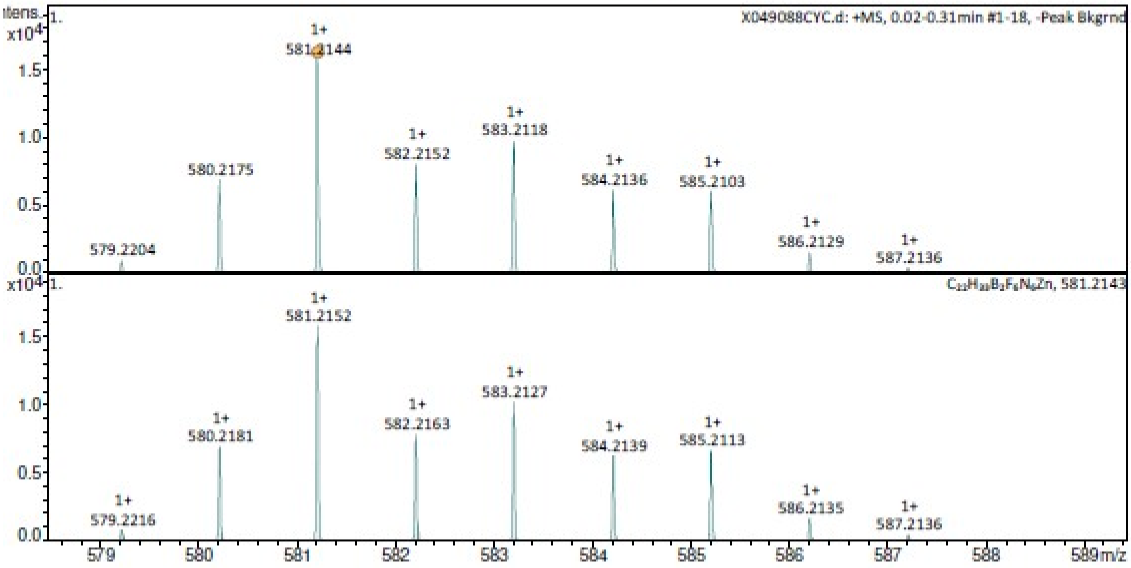

Meas. m/z	z	#	Ion Formula	m/z	err [ppm]	mSigma	rdb	e⁻ Conf
271.104737	2+	1	C22H32B2F4N6Zn	271.104557	1.0	43.6	10.0	even
561.208285	1+	1	C22H32B2F5N6Zn	561.208066	1.2	51.5	9.0	even
581.214445	1+	1	C22H33B2F6N6Zn	581.214294	1.3	16.6	8.0	even
603.196279	1+	1	C22H32B2F6N6NaZn	603.196238	1.4	28.2	8.0	even
619.170832	1+	1	C22H32B2F6KN6Zn	619.170175	0.4	58.3	8.0	even

Institut de Chimie Organique et Analytique
UMR 7311 - Université d'Orléans - CNRS
BP 6759 - rue de Chartres - F-45067 ORLEANS Cedex 2
cyril.colas@univ-orleans.fr
+33 (0)2 38 49 46 61
printed: 12/04/2019 12:56:09
Page 1 of 1

X-Ray diffraction data:

$H_2Do2py2BF_3$ (pH 4)

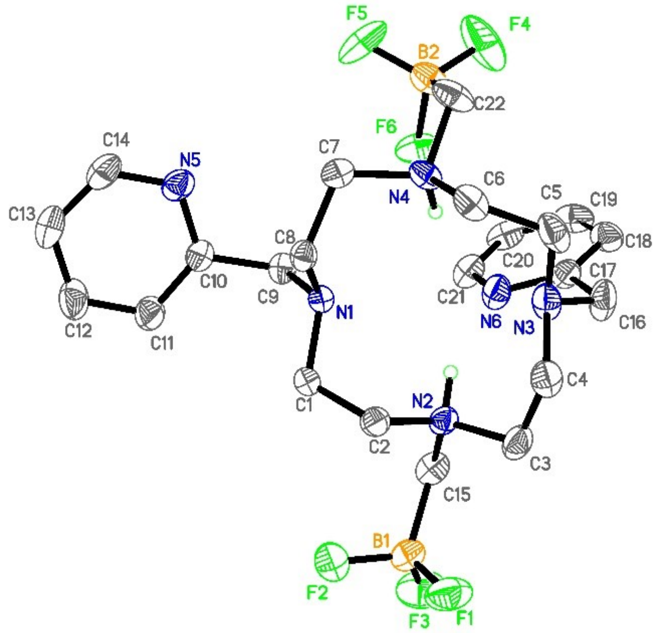

molecule

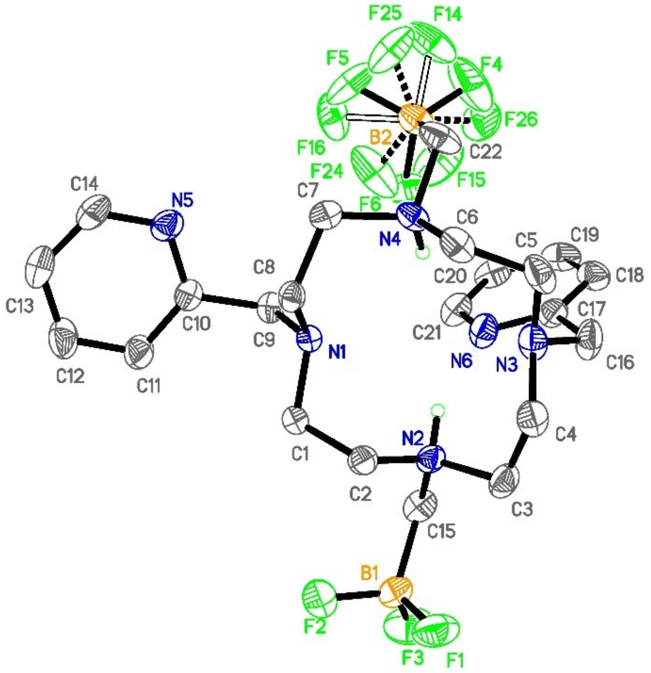

asymmetric unit

Table S1 - Crystal data and structure refinement for $H_2Do2py2BF_3$ (pH 4)

Identification code	H_2Do2py2BF3	
Empirical formula	C22 H34 B2 F6 N6	
Formula weight	518.17	
Temperature	193(2) K	
Wavelength	0.71073 Å	
Crystal system	Monoclinic	
Space group	*P* 2_1*/c*	
Unit cell dimensions	a = 11.5578(8) Å	α= 90°.
	b = 15.0808(10) Å	β= 109.789(2)°.
	c = 15.1865(8) Å	γ = 90°.
Volume	2490.7(3) $Å^3$	
Z	4	
Density (calculated)	1.382 Mg/m^3	
Absorption coefficient	0.115 mm^{-1}	
F(000)	1088	
Crystal size	0.700 x 0.200 x 0.160 mm^3	
Theta range for data collection	3.029 to 28.754°.	
Index ranges	-15<=h<=15, -20<=k<=20, -20<=l<=17	
Reflections collected	108499	
Independent reflections	6459 [R(int) = 0.0492]	
Completeness to theta = 25.242°	99.8 %	
Absorption correction	Semi-empirical from equivalents	
Max. and min. transmission	0.7458 and 0.6753	
Refinement method	Full-matrix least-squares on F^2	
Data / restraints / parameters	6459 / 126 / 387	
Goodness-of-fit on F^2	1.011	
Final R indices [I>2sigma(I)]	R1 = 0.0511, wR2 = 0.1152	
R indices (all data)	R1 = 0.0752, wR2 = 0.1321	
Extinction coefficient	n/a	
Largest diff. peak and hole	0.399 and -0.333 e.$Å^{-3}$	

$H_4Do2py2BF_3^{2+}$ (pH 1)

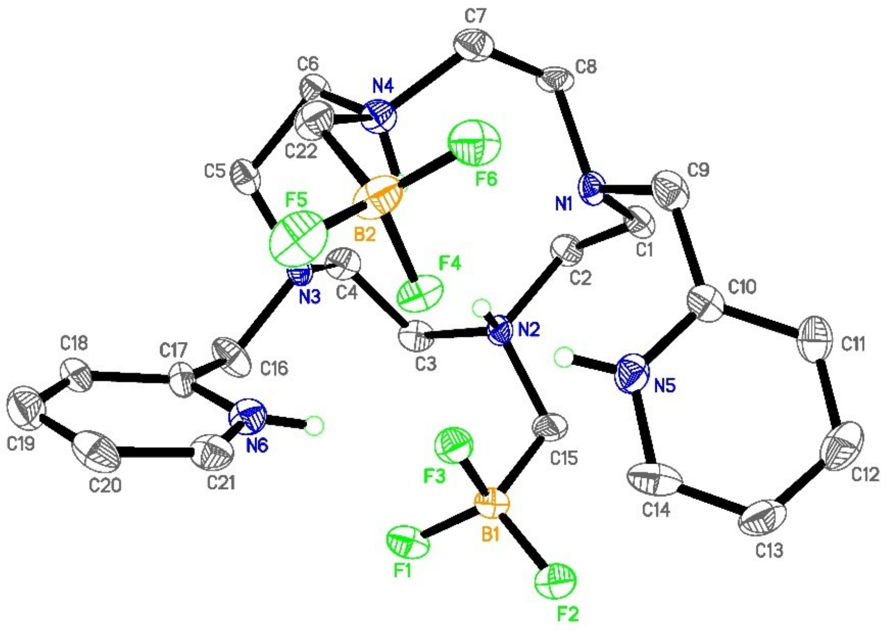

cationic part of the structure

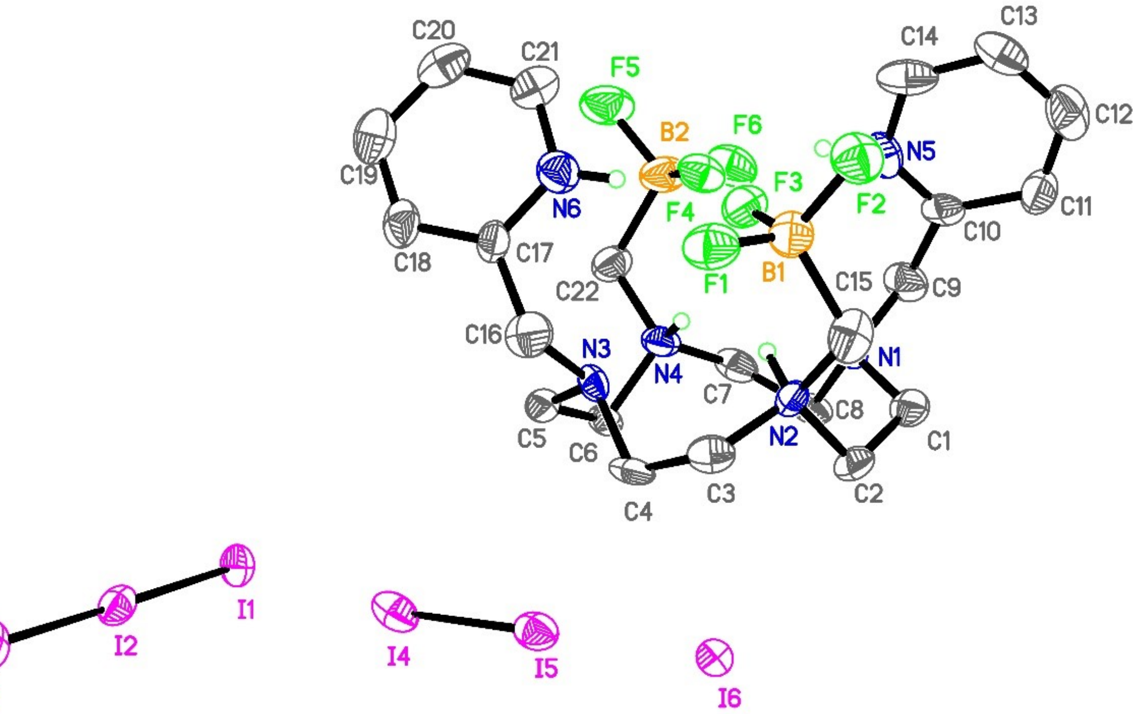

asymmetric unit

Table S2 - Crystal data and structure refinement for $H_4Do2py2BF_3^{2+}$ (pH 1)

Identification code	$H_4Do2py2BF_3^{2+}$	
Empirical formula	C22 H36 B2 F6 N6, I6	
Formula weight	1281.59	
Temperature	193(2) K	
Wavelength	1.54178 Å	
Crystal system	Triclinic	
Space group	$P\ \bar{1}$	
Unit cell dimensions	a = 12.8808(5) Å	α= 75.784(3)°.
	b = 12.9692(5) Å	β= 67.305(3)°.
	c = 13.6306(5) Å	γ = 60.961(3)°.
Volume	1831.86(13) Å^3	
Z	2	
Density (calculated)	2.323 Mg/m^3	
Absorption coefficient	40.430 mm^{-1}	
F(000)	1184	
Crystal size	0.060 x 0.050 x 0.020 mm^3	
Theta range for data collection	3.524 to 66.770°.	
Index ranges	-15<=h<=15, -15<=k<=15, -10<=l<=16	
Reflections collected	6486	
Independent reflections	6486 [R(int) = ?]	
Completeness to theta = 66.770°	99.6 %	
Absorption correction	Semi-empirical from equivalents	
Max. and min. transmission	0.7528 and 0.3939	
Refinement method	Full-matrix least-squares on F^2	
Data / restraints / parameters	6486 / 1 / 392	
Goodness-of-fit on F^2	1.002	
Final R indices [I>2sigma(I)]	R1 = 0.0541, wR2 = 0.1187	
R indices (all data)	R1 = 0.0984, wR2 = 0.1381	
Extinction coefficient	n/a	
Largest diff. peak and hole	1.491 and -1.315 e.Å^{-3}	

Zn($Do2py2BF_3$) co

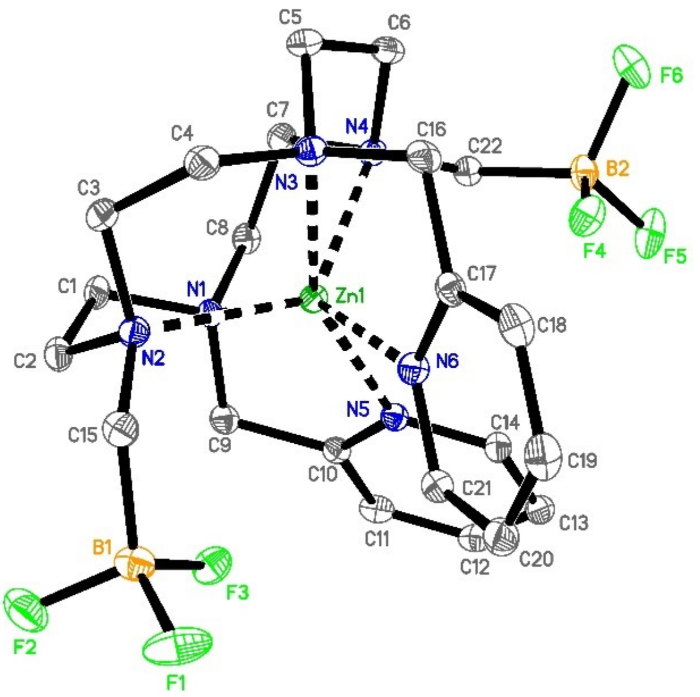

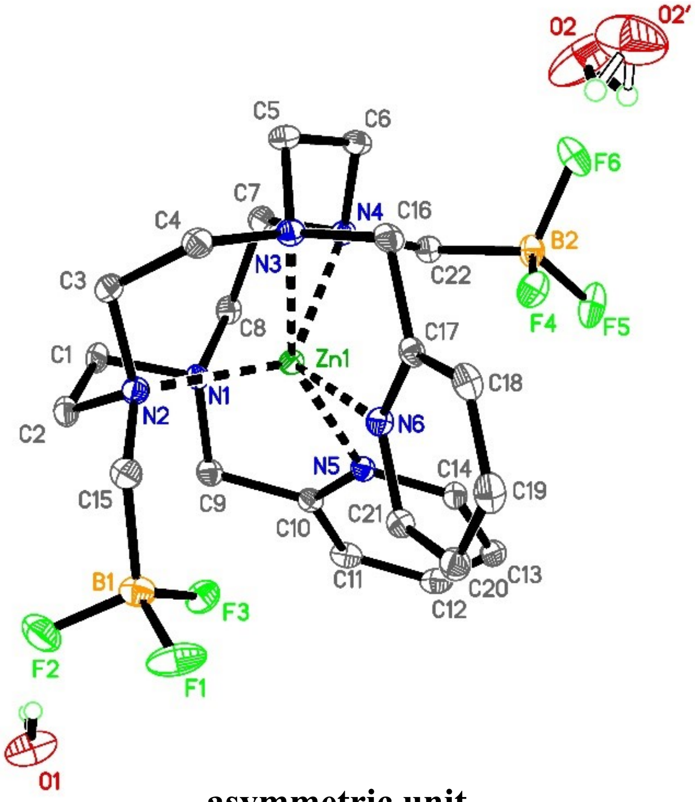

asymmetric unit

Table S3 - Crystal data and structure refinement for Zn(Do2py2BF_3)

Identification code	Zn(Do2py2BF3)	
Empirical formula	C22 H32 B2 F6 N6 Zn, 2 H2 O,	
Formula weight	617.56	
Temperature	193(2) K	
Wavelength	0.71073 Å	
Crystal system	Orthorhombic	
Space group	*P ca2$_1$*	
Unit cell dimensions	a = 17.1147(8) Å	α= 90°.
	b = 9.0627(5) Å	β= 90°.
	c = 16.9949(8) Å	γ = 90°.
Volume	2636.0(2) $Å^3$	
Z	4	
Density (calculated)	1.556 Mg/m^3	
Absorption coefficient	1.008 mm^{-1}	
F(000)	1280	
Crystal size	0.250 x 0.200 x 0.050 mm^3	
Theta range for data collection	3.274 to 28.310°.	
Index ranges	-22<=h<=22, -12<=k<=12, -19<=l<=22	
Reflections collected	64889	
Independent reflections	6090 [R(int) = 0.0376]	
Completeness to theta = 25.242°	99.8 %	
Absorption correction	Semi-empirical from equivalents	
Max. and min. transmission	0.7457 and 0.6317	
Refinement method	Full-matrix least-squares on F^2	
Data / restraints / parameters	6090 / 7 / 376	
Goodness-of-fit on F^2	1.047	
Final R indices [I>2sigma(I)]	R1 = 0.0239, wR2 = 0.0499	
R indices (all data)	R1 = 0.0303, wR2 = 0.0521	
Absolute structure parameter	0.006(3)	
Extinction coefficient	n/a	
Largest diff. peak and hole	0.224 and -0.319 e.$Å^{-3}$	

NMR monitoring of $Do2py2BF_3$ and $Zn(Do2py2BF_3)$ pH-dependant stability

3 pH conditions were used to assess stability of **$Do2py2BF_3$** ligand and **$Zn(Do2py2BF_3)$**, at concentrations of ca. 3.10^{-2} M in aqueous buffered solution with addition of DMSO to ensure full solubility of studied molecules as well as eventual degradation products. Monitoring was carried out by ^{19}F NMR with a minimum of 6 timepoints between. Trifluoroacetic acid (TFA) in solution in water was added in NMR tubes in a sealed capillary tube as an internal reference.

- Acidic pH (2 ± 0.2): HCl in H_2O (0.5 mL) + DMSO (0.4 mL).
- Neutral pH (7.3 ± 0.2): Acetate buffer (0.5 mL) + DMSO (0.4 mL).
- Basic pH (9.7 ± 0.2): Ammonia buffer (0.5 mL) + DMSO (0.4 mL).

Figure S15: $H_2Do2py2BF_3$ ligand (pH 2, 25°C)

Nb: at acidic pH, ligand is under the form $H_4Do2py2BF_3^{2+}$, with three broad ^{19}F signals (vide supra). Signals are slightly shifted owing to the different solvent system (D_2O/DMSO vs. D_2O only) but confirms the multiplicity and shape of the signals corresponding to the intact ligand from control spectrum.

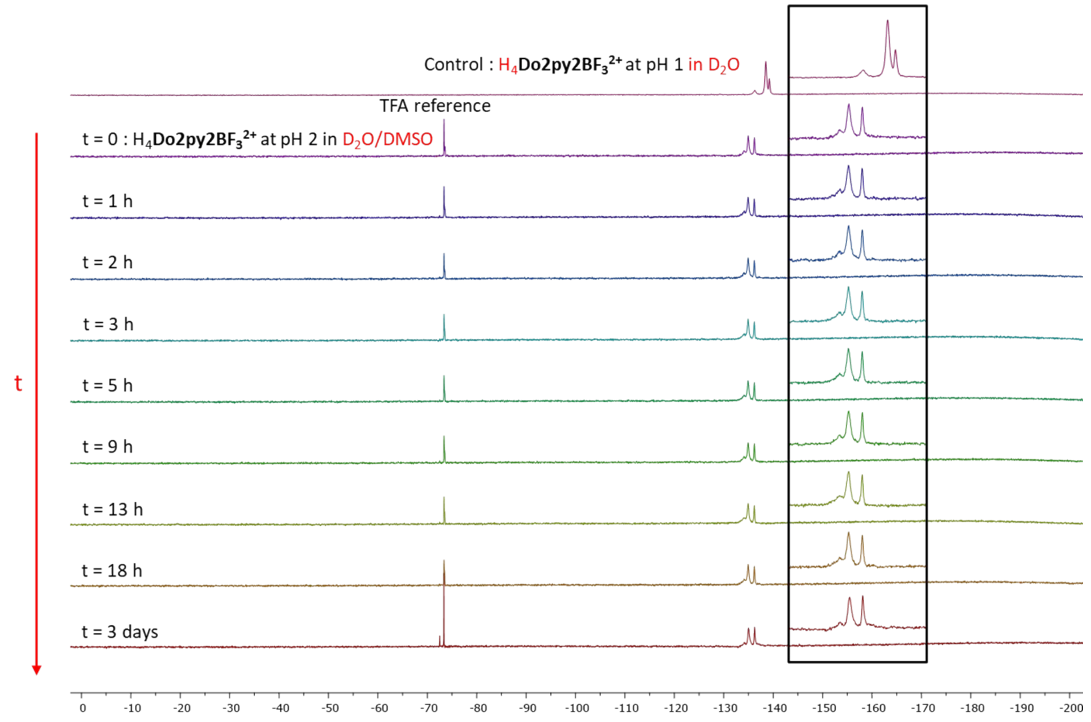

Figure S16: H_2Do2py2BF$_3$ ligand (pH 7.3)

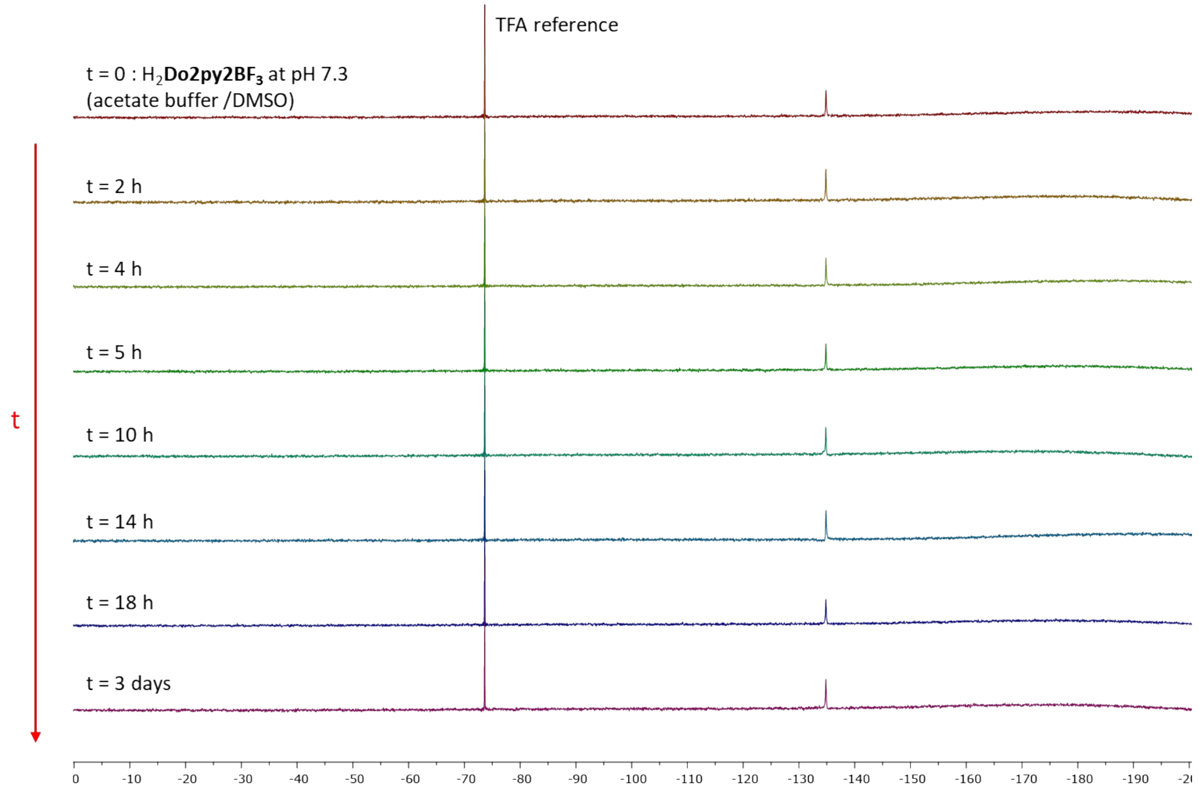

Figure S17: H_2Do2py2BF$_3$ ligand (pH 9.7)

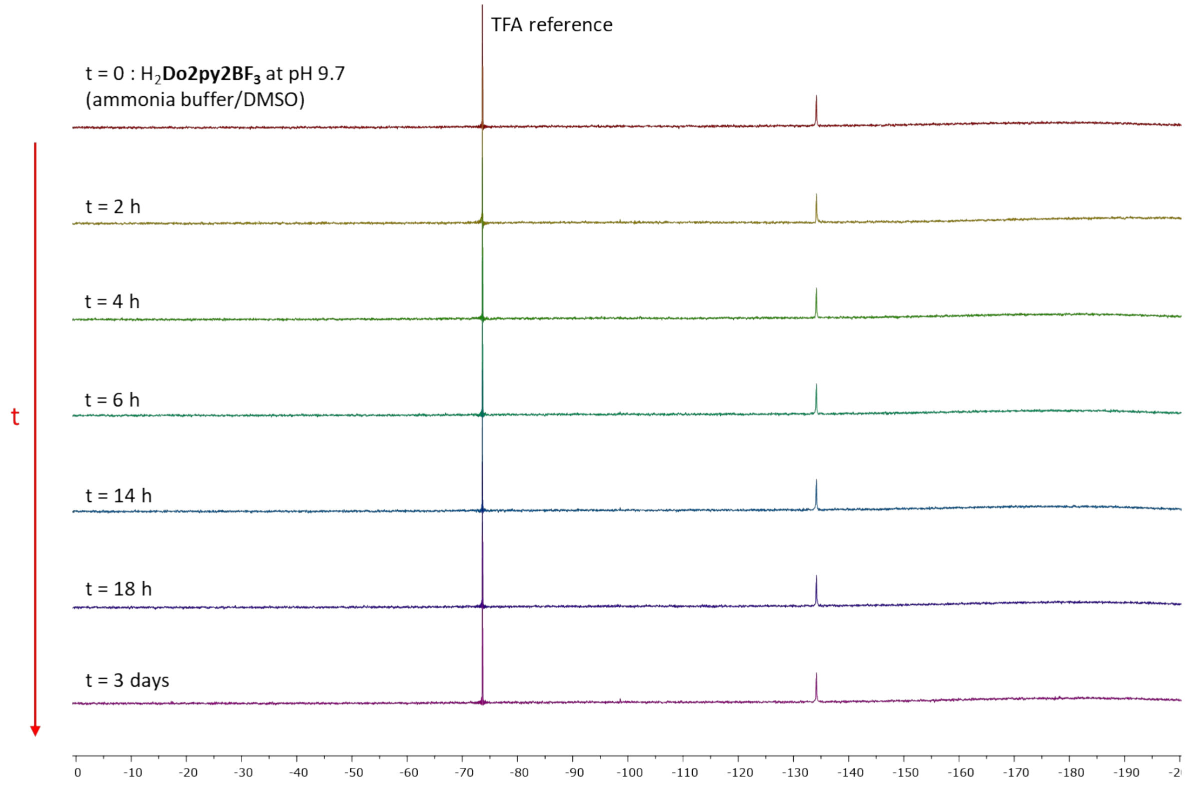

Figure S18: Zn(Do2py2BF$_3$) complex (pH 2)

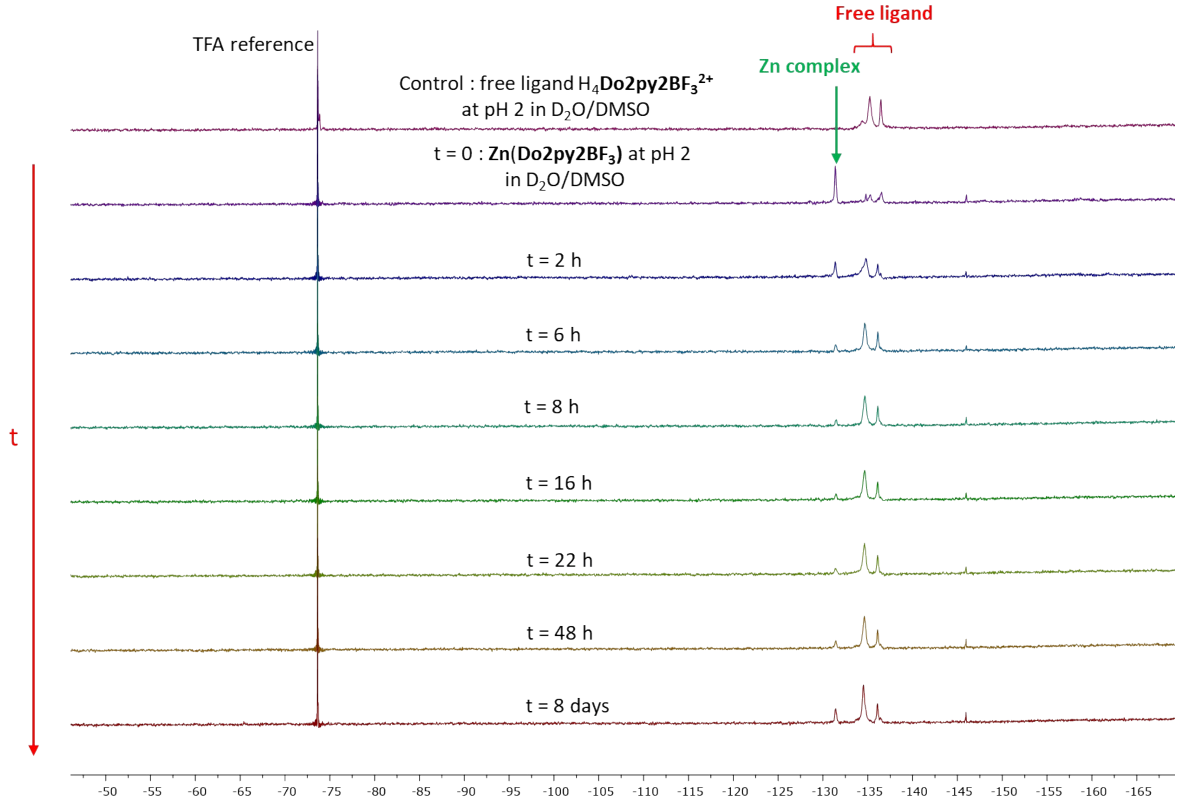

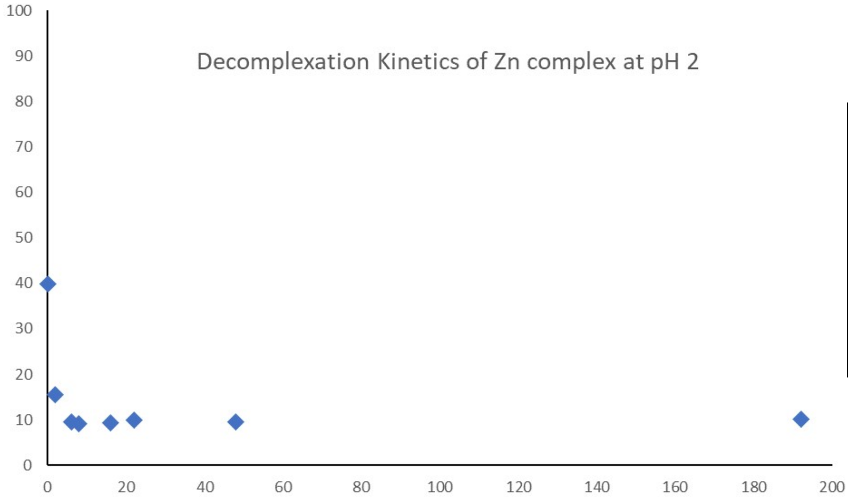

t(hours)	complex/(total 19F) ratio
0	39,8
2	15,6
6	9,5
8	9,1
16	9,3
22	9,9
48	9,6
192	10,1

Data from integration of ^{19}F spectrum with TFA (capillary) as reference

Nb : t=0 corresponds to the first ^{19}F NMR measurement which is <5 minutes when taking into account the time required for spectrometer setting + acquisition time. $t_{1/2}$ can therefore only be estimated as < 5 minutes.

Figure S19: $Zn(Do2py2BF_3)$ complex (pH 7.3)

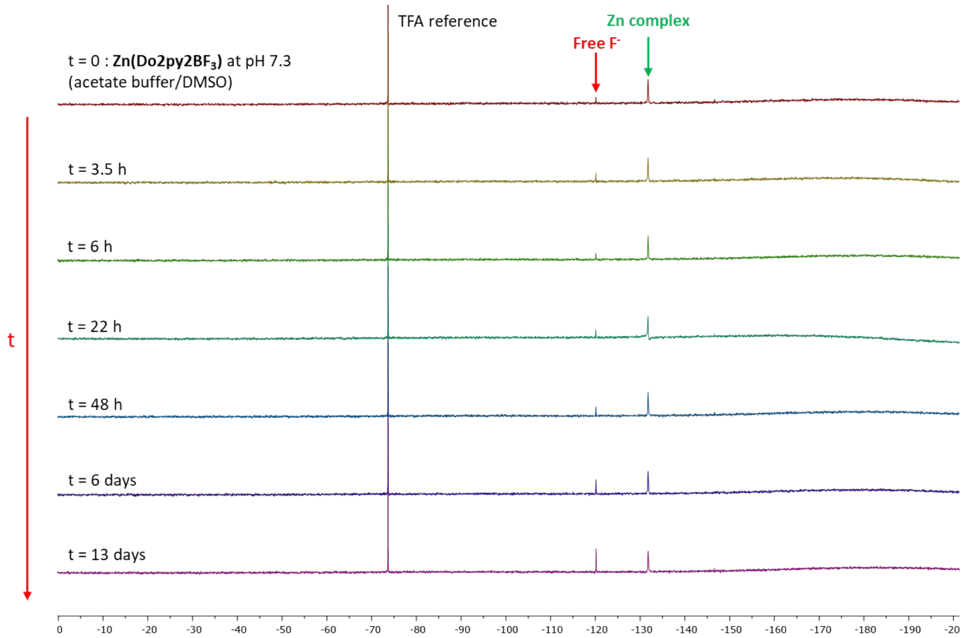

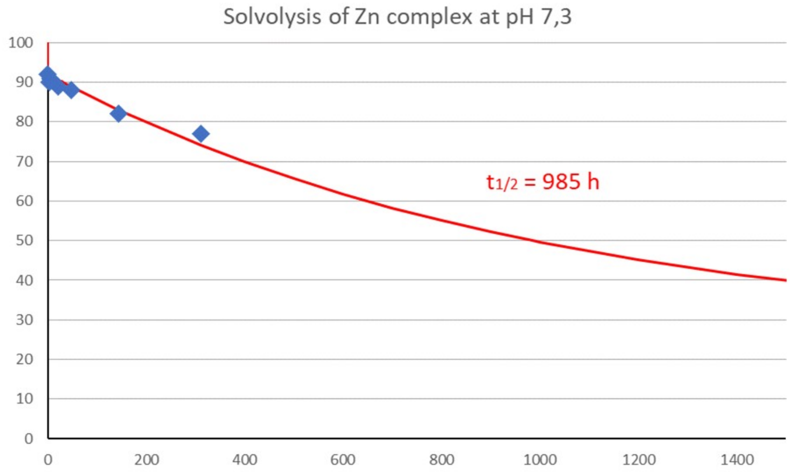

Data from integration of ^{19}F spectrum with TFA (capillary) as reference

t(hours)	complex/(total 19F) ratio
0	92
3,5	90
6	91
22	89
48	88
144	82
312	77

Figure S20: $Zn(Do2py2BF_3)$ complex (pH 9.7)

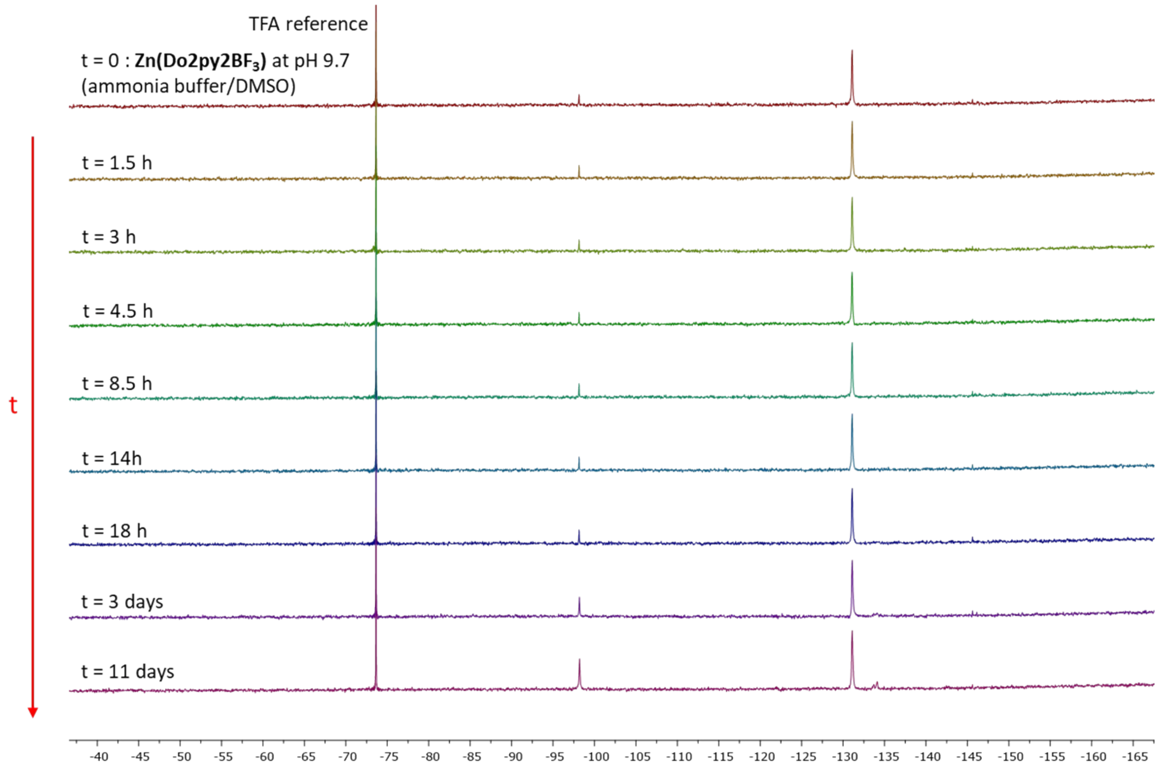

t(hours)	complex/(total 19F) ratio
0	95
1,5	94
3	91
4,5	92
8,5	91
13,5	91
18	90
72	83
264	63

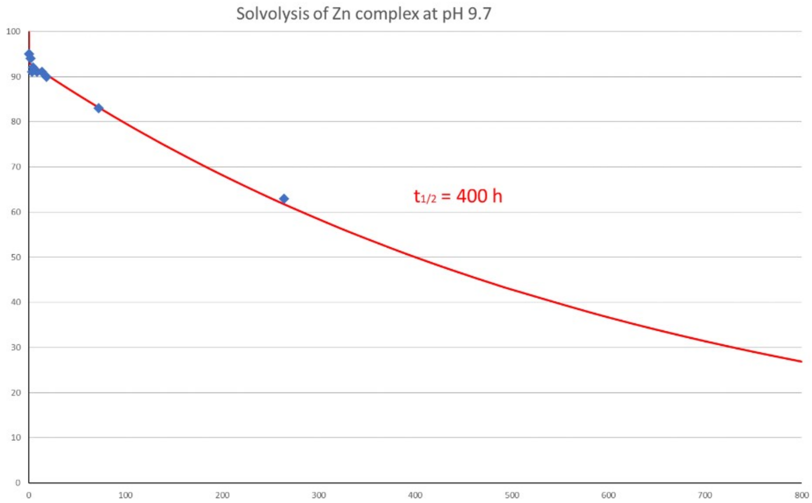

Data from integration of ^{19}F spectrum with TFA (capillary) as reference

^{18}F-Radiolabeling

All solvents and reagents were purchased from commercial suppliers, namely Sigma Aldrich or VWR, and were used as received without further purification. Sep-Pak® Accell Plus QMA Plus Light Cartridges were purchased from Waters Corporation and were pre-conditioned with 2 ml of TBABr (10 mg/mL in H_2O) and rinsed with 18 mL of water.

[^{18}F] fluoride was produced by CYROI via the $^{18}O(p,n)^{18}F$ nuclear reaction with a GE cyclotron (PETtrace 800, 16.5 MeV). The bombardment of ^{18}O-enriched water with protons at 80 µA during 15 min provided about 30 GBq of [^{18}F]-fluoride in ^{18}O-enriched water (4 mL), then the activity was directly transferred under helium pressure to the radiosynthesis automate. A remote controlled GE TracerLab FX FN module was used for the automated radiolabeling experiments. Helium was used as a pressurizing gas during sample transfers.

For the RadioChemical Yields (RCY) measurements, the HPLC analysis were performed on a UPLC Ultimate 3000 system (Thermo fisher Scientific) equipped with a UV spectrophotometer and a Berthold Flowstar LB 514 radioactivity detector, column: Gemini C18, 250 × 4.6 mm, 5µm (Phenomenex), Elution condition : NH_4OAc 0.1M (pH 10) (A) /MeCN (B) from 0 to 1 min 90/10 (A/B); a gradient from 1 min to 21 min to reach 20/80 (A/B) until 25 min, then to 26 min 90/10 (A/B); flow rate: 1 mL/min; room temperature, UV detection λ=258 nm.

Automated radiosynthesis

After the end of bombardment (EOB), the [^{18}F] fluoride (30 GBq) was delivered to the automate and trapped on a pre-conditioned Sep-Pak Accell Plus QMA Plus Light Cartridge to remove ^{18}O-enriched water and others impurities. The whole activity was released from the cartridge into the reaction vessel by the aqueous solution of TBABr (16 mg in 0.5 mL) in vial 1 to form [^{18}F]tetrabutylammonium fluoride ([^{18}F]TBAF). 1 mL of acetonitrile from vial 2 was poured into the reaction vessel to provide anhydrous [^{18}F]TBAF thanks to an azeotropic distillation under vacuum and helium flow by heating at 100°C. A solution containing the 1 mg of ligand $H_2Do_2py_2BF_3$ in 1 ml acetonitrile or $Zn(Do_2py_2BF_3)$ in 1mL DMSO was poured into the reactor by applying helium pressure on vial 3. The mixture was stirred for 30 min at 80°C. The resulting solution was sucked into a vial. A sample was taken for analysis. The HPLC vial was measured into a shielded glove box with dose calibrator Scintidose (Lemer Pax) before and after the HPLC injection (10µL).

Figure S21: Scheme of TRACERLab FX FN module.

V1: 16 mg of TBABr in 0.5 mL water. V2: 1 mL of acetonitrile. V3: 1 mg of ligand in 1 mL of acetonitrile or DMSO

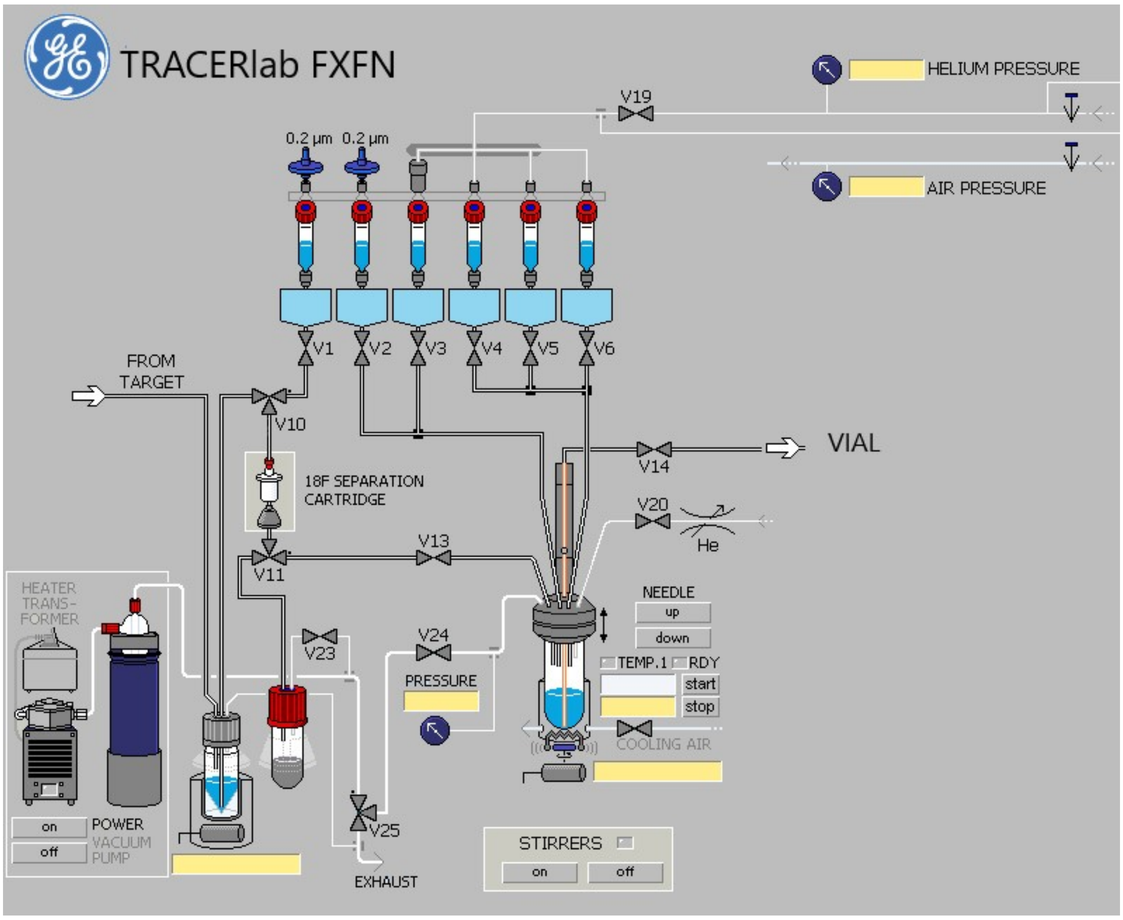

Figure S22: HPLC analysis of $H_2Do2Py2BF_3$ radiolabeling.

Elution condition: NH4OAc 0.1M (pH 10) (A) /MeCN (B) from 0 to 1 min 90/10 (A/B); a gradient from 1 min to 21 min to reach 20/80 (A/B) until 25 min, then to 26 min 90/10 (A/B); flow rate: 1 mL/min; room temperature, UV detection λ=258 nm and radioactivity detector (Berthold Flowstar LB 514).

[1] $H_2Do2Py2BF_3$ 0.5 mg/mL in ACN (UV 258 nm).

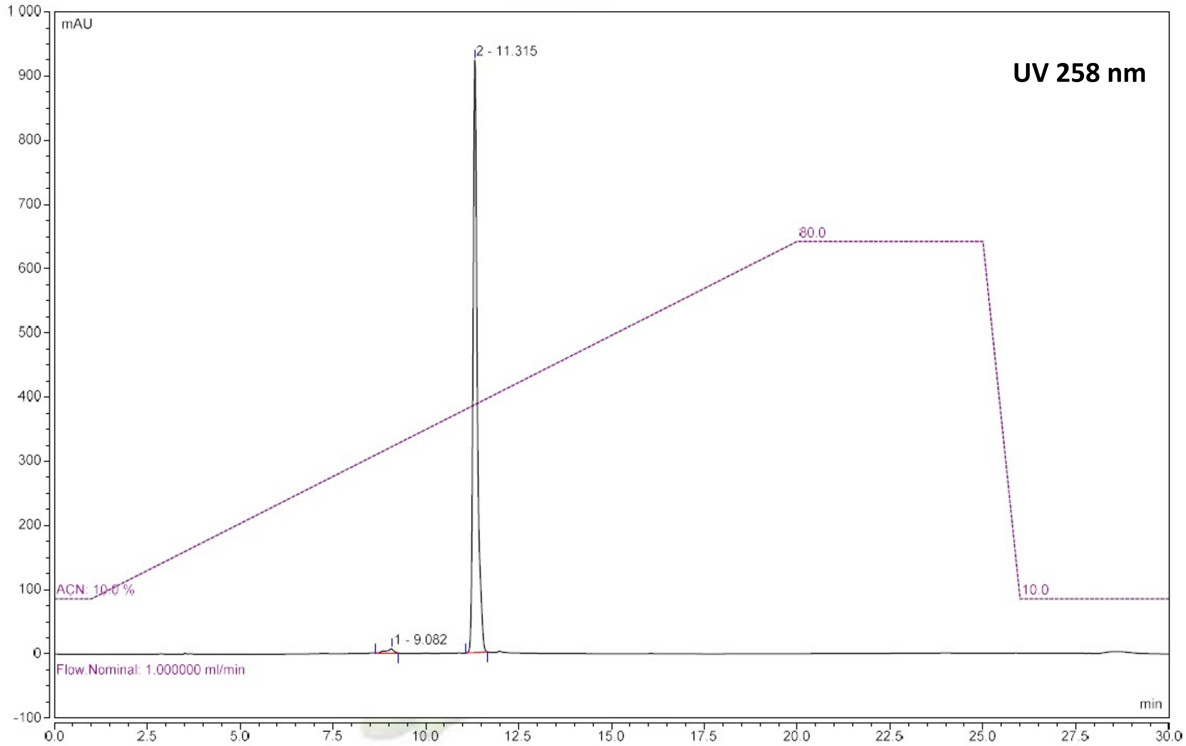

[2] Radio spectrum of [18F] $H_2Do2Py2BF_3$ in ACN.

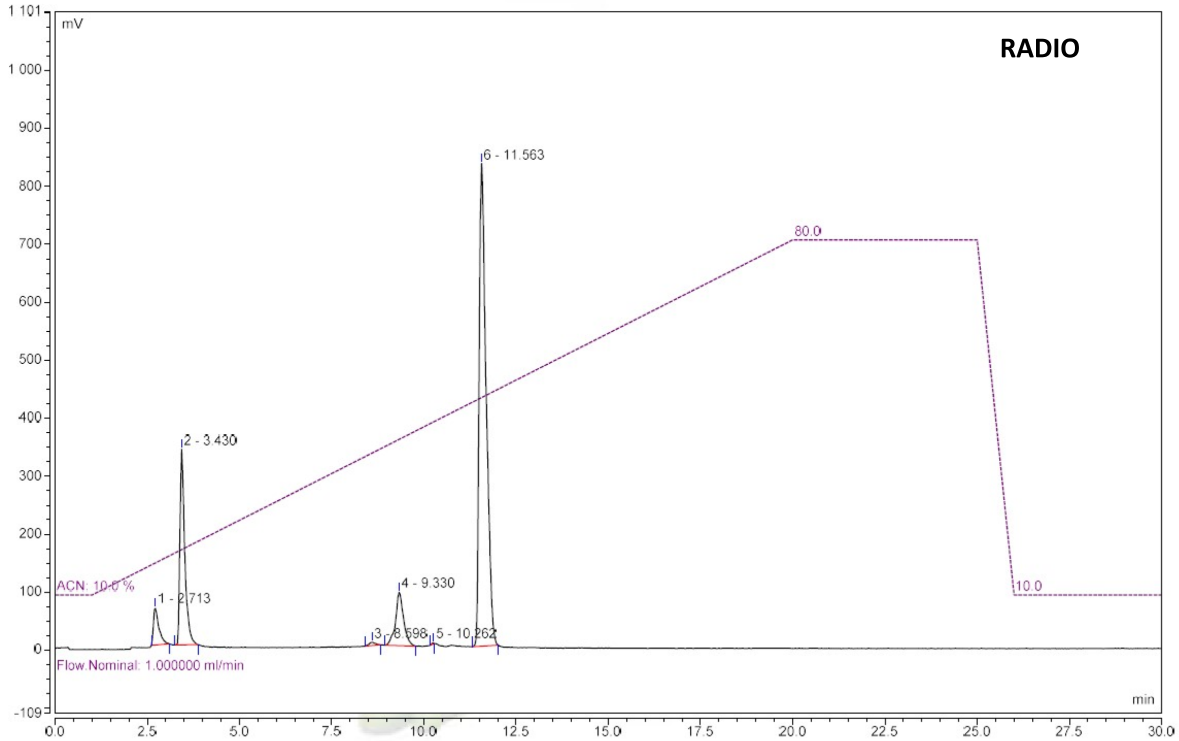

[3] UV spectrum of [18F] $H_2Do2Py2BF_3$ in ACN

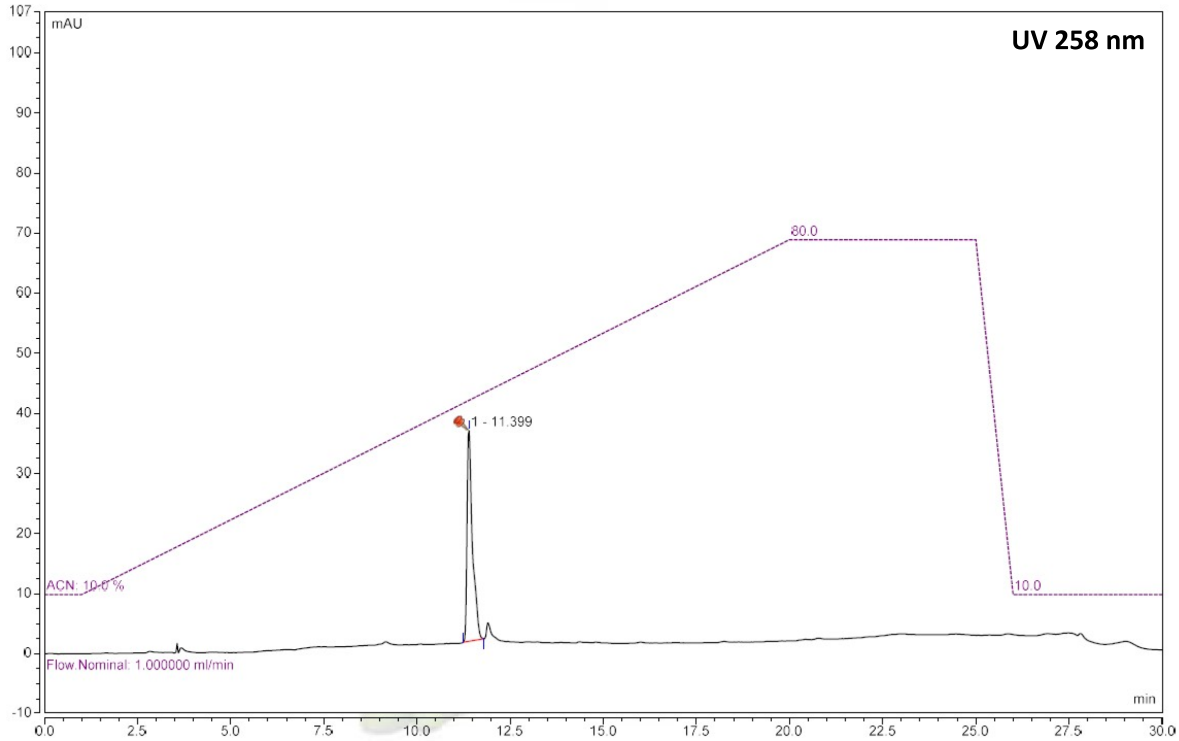

Figure S23: HPLC analysis of Zn(Do2Py2BF_3) radiolabeling.

Elution condition: NH4OAc 0.1M (pH 10) (A) /MeCN (B) from 0 to 1 min 90/10 (A/B); a gradient from 1 min to 21 min to reach 20/80 (A/B) until 25 min, then to 26 min 90/10 (A/B); flow rate: 1 mL/min; room temperature, UV detection λ=258 nm and radioactivity detector (Berthold Flowstar LB 514).

[1] Zn-$Do_2Py_2BF_3$ 0.5 mg/mL in DMSO (UV 258 nm);

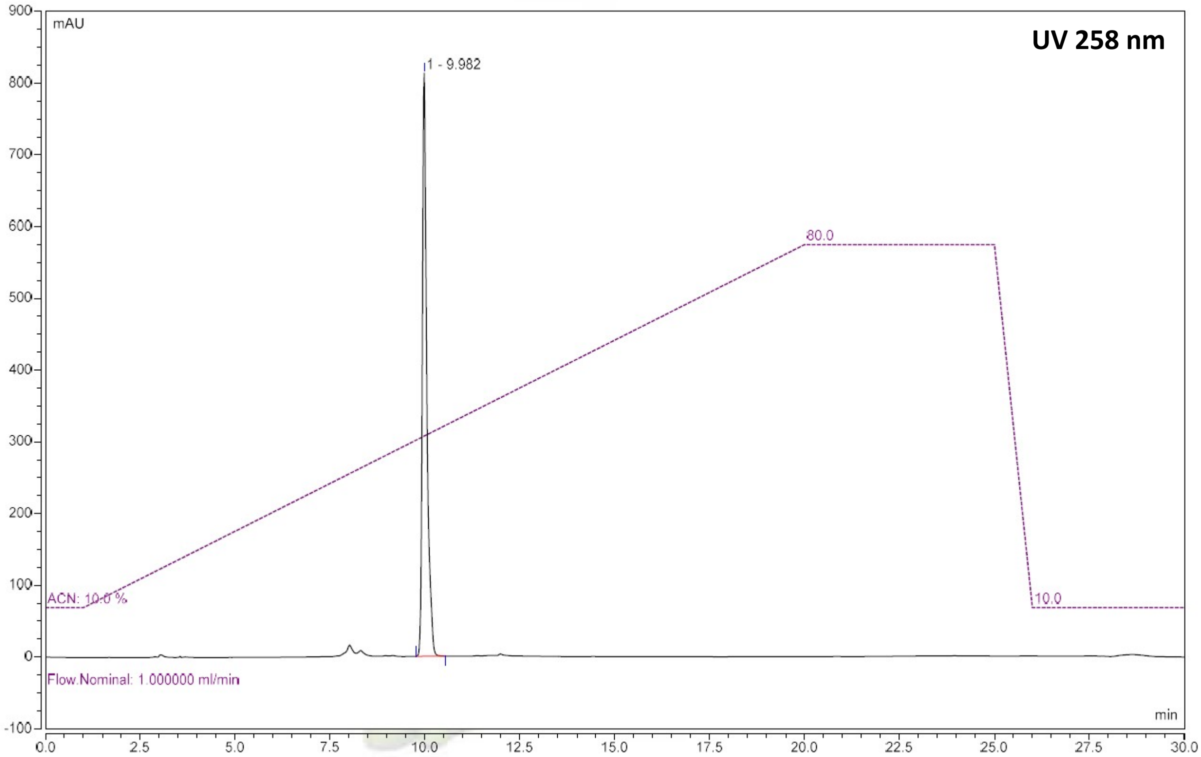

[2] Radio spectrum of [^{18}F] Zn-Do$_2$Py$_2$BF$_3$ in DMSO

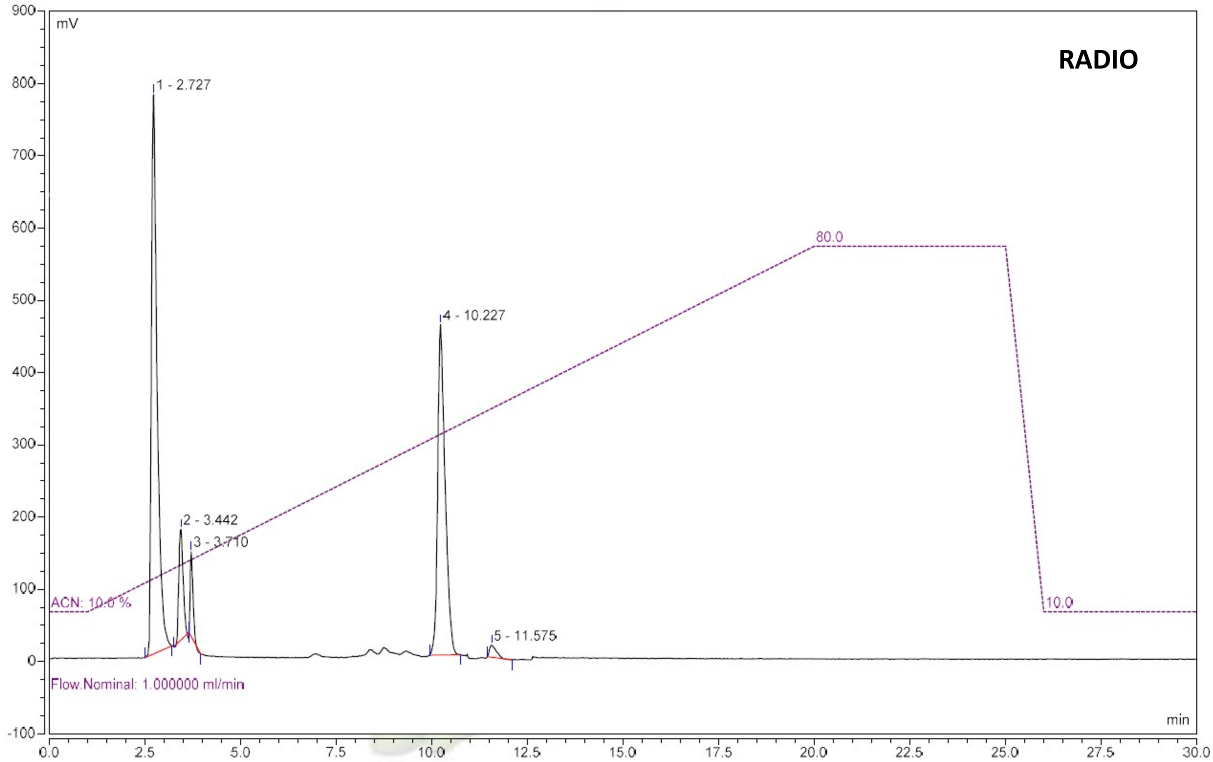

[3] UV spectrum of [^{18}F] Zn-Do$_2$Py$_2$BF$_3$ in DMSO.

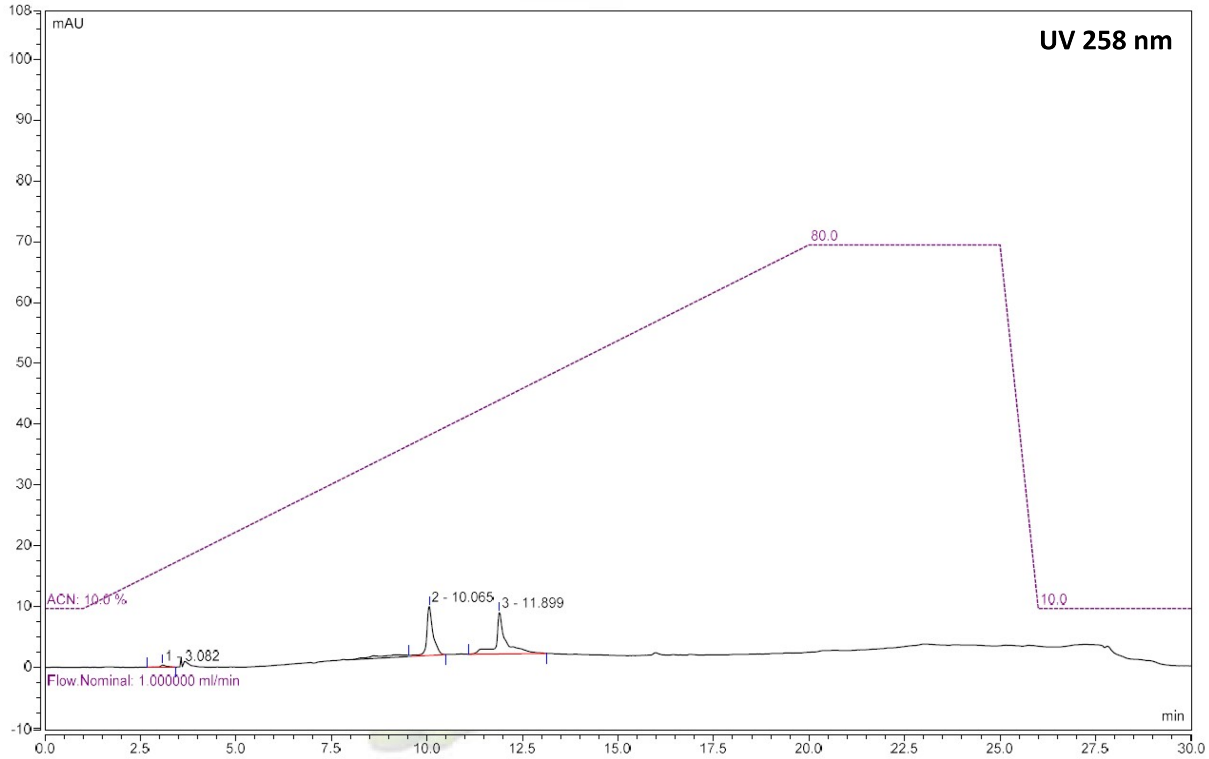

^{19}F-Magnetic Resonance

Zn(Do2py2BF$_3$) was dissolved in CH_3CN:H_20 (1:1) at a concentration of 18.5 mg/mL (31.8 mM, 190.8 mM in 19F).

1H MRI: Rare sequence, Repetition Time (TR) = 4145 ms, Echo Time (TE) = 7.67 ms, Rare Factor (RF) = 32, Number of Averages (NAV) = 4, Field of View (FOV) = 35 x 35 mm, Matrix = 256 x 256, Slice Thickness = 2 mm, Acquisition Time = 2 min 12 s.

19F MRI: Rare sequence, TR = 2000 ms, TE = 38.1 ms, RF = 24, FOV = 35 x 35 mm, Matrix = 32 x 32, Slice Thickness = 2 mm, NAV = 300, 1350 or 1800 corresponding to an Acquisition Time of = 10, 45 or 60 min respectively, o1p = -134.5 ppm, Spectral width = 20000 Hz. The acquired image was then reconstructed with a 256 x 256 matrix (bicubic interpolation) in order to superimpose it to the 1H MR image. Image analysis was carried out with ImageJ: selected Regions of Interest (ROIs) were drawn on the different samples of the phantom and the mean signal intensity in each sample was measured. Then the SI was plotted against the 19F concentration values.

Figure S24: ^{19}F Signal Intensity values at 7T

Signal intensity determined by ^{19}F MRI reported against ^{19}F mM concentration (N AV 1800, Acq Time 60 min, Slice Thickness 2 mm, Matrix 32x32).

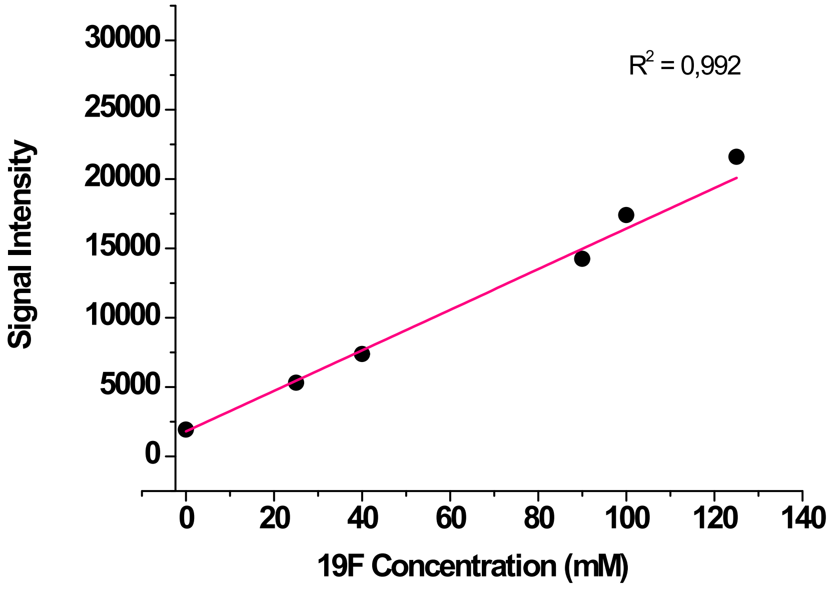

References

[1] J.-F. Morfin, R. Tripier, M. L. Baccon, H. Handel, *Polyhedron* **2009**, *28*, 3691–3698.

[2] N. Bernier, J. Costa, R. Delgado, V. Félix, G. Royal, R. Tripier, *Dalton Trans.* **2011**, *40*, 4514–4526.
